# Supplementary material for: A computational model for angular velocity integration in a locust heading circuit
Source: PLoS Comput Biol. 2024 Dec 20;20(12):e1012155. doi: 10.1371/journal.pcbi.1012155 (PMC11703117; doi:10.1371/journal.pcbi.1012155)
Supplement: S1 Text — (PDF) [file pcbi.1012155.s001.pdf]

S1 Text: Neuron model derivation

The network was modelled based on single-compartment steady-state firing rate neurons abstracted from integrate-and-fire neurons [1]. We outline the abstraction process in the following.

All potentials are measured relative to the membrane resting potential. Let U be the membrane potential, and c_m and g_m the specific membrane capacitance and conductance, respectively. For a neuron with synapses indexed by i, $g_{s,i}$ denotes the specific synaptic conductance of synapse i, which we refer to as its weight. $P_{s,i}$ is the synaptic channel opening probability, and $E_{s,i}$ is the synaptic reversal potential. The time course of U is then governed by the differential equation (cf. Equation 5.7 in [1]):

$$c_m \frac{dU}{dt} = -g_m U - \sum_i g_{s,i} P_{s,i} (U - E_{s,i}) \tag{1}$$

We divide on both sides by g_m, introduce the membrane time constant $\tau_m = \frac{c_m}{g_m}$ and the relative synaptic weights $\tilde{g}_{s,i} = \frac{g_{s,i}}{g_m}$ (cf. Equation 5.43 from [1]):

$$\tau_m \frac{dU}{dt} = -U - \sum_i \tilde{g}_{s,i} P_{s,i} (U - E_{s,i}) \tag{2}$$

We assume that the membrane time constant τ_m is at least an order of magnitude smaller than the synaptic time constant τ_s, which governs the dynamics of $P_{s,i}$. Estimates for τ_m vary with neuron type. While $\tau_m = 1.5\,\text{ms}$ can be derived from the resting state conductance of a Hodgkin-Huxley model with the parameters values given in [1], the membrane time constants used in the fruit fly CX model of [2] are larger by an order of magnitude to capture the observed delays between E-PG and P-EN activity in walking flies [2]. We chose to model these delays by slow synapses, i.e. $\tau_s \gg \tau_m$. This relationship between the time constants justifies a steady-state approximation to the membrane dynamics. We introduce the steady-state potential

$$U_\infty = \frac{\sum_i \tilde{g}_{s,i} P_{s,i} E_{s,i}}{1 + \sum_i \tilde{g}_{s,i} P_{s,i}} \tag{3}$$

which is reached for constant $P_{s,i}$ after waiting for a long enough time so that $\frac{dU}{dt} = 0$. Note that U_∞ also evolves in time, but on a slower scale than U. Using this definition, we can rewrite Equation 2 as

$$\tau_{ms} \frac{dU}{dt} = -(U - U_\infty) \tag{4}$$

where $\tau_{ms} = \frac{\tau_m}{1 + \sum_i \tilde{g}_{s,i} P_{s,i}}$ is the membrane time constant corrected for synaptic weights. Following the derivation in [1], we can then compute the firing rate r of an integrate-and-fire neuron whose membrane potential is governed by Equation 4 between spikes via

$$r(U_\infty) = \left[\tau_{ref} + \tau_{ms} \ln \left(\frac{U_\infty - U_{res}}{U_\infty - U_{th}} \right) \right]^{-1} \tag{5}$$

where τ_{ref} is the refractory period during which the membrane potential is held at the resetting potential U_{res} after a spike. U_{th} is the threshold potential: if $U \geq U_{th}$, a spike is triggered. Equation 5 is valid for a deterministic, noise-free neuron. However, membrane potential fluctuations will

lead to deviations whose average effect can be well described by a logistic sigmoid:

$$r(U_\infty) \approx \frac{A}{1 + \exp(-B \cdot (U_\infty - C))} \tag{6}$$

We used the following neuron parameters: $\tau_m = 1.5\,\mathrm{ms}$. Furthermore, $\tau_{ref} = 9\,\mathrm{ms}$, $U_{res} = -11\,\mathrm{mV}$, and $U_{th} = 15\,\mathrm{mV}$. As stated above, U_{res} and U_{th} are measured relative to the membrane resting potential of $\approx -65\,\mathrm{mV}$. We chose the values of U_{th} and τ_{ref} so that the spike times of the integrate-and-fire model are as similar as possible to the spike times of a Hodgkin-Huxley simulation with the parameter values in [1]. For random fluctuations with a standard deviation of 8 mV, which has been observed in locust neurons (see e.g. [3], Fig. 2), the best fitting sigmoid parameters are:

$$A = 99.6\,\mathrm{Hz},\ B = 0.19\,\mathrm{mV}^{-1}\ C = 17.8\,\mathrm{mV}. \tag{7}$$

These values can be reproduced by running the script `invertpy/brain/centralcomplex/cl1acl2/fit_rate_function.py` in the repository. This script will also output S2 Fig, which shows the logistic sigmoid approximation to the average rate function with 8 mV membrane noise.

Note that an average rate function with sigmoid shape is obtained for almost any zero mean noise with nonzero standard deviation, so the value of 8 mV is not critical.

An additional bias neuron innervates all columnar neurons. It always fires at a high rate (ca. 100 Hz) to maintain a stable operating point for CL1a- and CL2-neurons (ca. 25 Hz). Other approaches for setting an operating point, such as a change of membrane properties, would be conceivable, too, but are biologically under-constrained.

Synapse model

We describe the time course of the synaptic open probability $P_{s,i}$ by a single-exponential kernel located at the time of a spike t_{spike}

$$P_{s,i} = P_{s,max} \exp\left(-\frac{t - t_{spike}}{\tau_s}\right) \tag{8}$$

where the synaptic time constant τ_s is typically in the range between 10 to 100 ms. Precise values for τ_s in the locust are currently unknown, but drawing on our estimates from available fruit fly data, we argue for moderately slow synapses (see supporting information S2 Text: Functional transmission delays between CL1a- and CL2-neuron populations). We chose $\tau_s = 20\,\mathrm{ms}$. This choice provides a balance between two important factors. First, it helps smooth out noise in angular velocity integration by acting as a low-pass filter. Second, it allows the compass representation to respond effectively to angular velocity inputs, within the range we have modeled: $[-150\,°/\mathrm{s}, 150\,°/\mathrm{s}]$.

Since our network is comprised of rate neurons, individual spike times are not available. We therefore describe the spike timing probability by an inhomogeneous Poisson process parameterised by the (time varying) presynaptic rate $r_i(t)$ of synapse i. Following the derivation in [1], the time course of $P_{s,i}$ can then be described by the differential equation

$$\frac{dP_{s,i}}{dt} = P_{s,max} r_i(t) - \left(r_i(t) + \tau_s^{-1}\right) P_{s,i} \tag{9}$$

where $P_{s,max}$ is the maximum synaptic open probability, which we set to 1.

We argue that the Poisson assumption is approximately valid, since r_i does not exceed 50 Hz in available data [4]. Therefore, effects of refractoriness on the regularity of the spike train can be compensated by small conductance changes. To demonstrate that this is the case, we connected two integrate-and-fire neurons with an excitatory synapse, using $\tau_s = 20$ ms. We simulated neurons with $\tau_m = 1.5$ ms and $t_{ref} = 9$ ms as in the manuscript, and a relative synaptic conductance $\tilde{g}_{tot} = 0.5$. We simulated membrane noise with a standard deviation of 8 mV over a 20 ms time period. The first neuron was driven by a sinusoidal input current with a frequency of 150 °/s and a magnitude chosen to cause a rate variation similar to that in our model with the fastest angular velocity input. We plotted an exemplary trace of the input current Ie_{n1}, the spikes and membrane potentials U of the two neurons n_1 and n_2, and the post-synaptic open probability $P_{n_1 \to n_2}$ in S3 Fig.

We repeated this simulation 1000 times, computed spike rate histograms across simulations and evaluated the corresponding rate model with a Poisson synapse, using the sigmoid activation function from the manuscript, see S4 Fig.

The top panel shows the rate of neuron n_1, the bottom panel neuron n_2. A good match between the two rate predictions can be observed. To achieve this match, we had to set the the Poisson synapse's $\tilde{g}_{tot} = 0.69$, i.e. slightly larger than in the integrate-and-fire model. This underscores our argument that the Poisson assumption does not invalidate our model in the presence of refractoriness. The conductance changes necessary for the intended behavior of the network will automatically be selected by our machine learning approach.

References

[1] Dayan P, Abbott LF. Theoretical Neuroscience. Computational and Mathematical Modeling of Neural Systems. Cambridge, MA: The MIT Press; 2001.

[2] Turner-Evans D, Wegener S, Rouault H, Franconville R, Wolff T, Seelig JD, et al. Angular velocity integration in a fly heading circuit. eLife. 2017;6:e23496. doi:https://doi.org/10.7554/eLife.23496.001.

[3] Moreaux L, Laurent G. Estimating firing rates from calcium signals in locust projection neurons *in vivo*. Frontiers in Neural Circuits. 2007;1. doi:https://doi.org/10.3389/neuro.04.002.2007.

[4] Zittrell F, Pabst K, Carlomagno E, Rosner R, Pegel U, Endres DM, et al. Integration of optic flow into the sky compass network in the brain of the desert locust. Frontiers in Neural Circuits. 2023;17:1111310. doi:https://doi.org/10.3389/fncir.2023.1111310.

S2 Text: Functional transmission delays between CL1a- and CL2-neuron populations

S2 Fig Sigmoid rate function approximation. Green dotted line: rate function of the ideal integrate-and-fire neuron without noise. Blue

dashed line: rate function with $\sigma = 8\,\mathrm{mV}$ membrane noise, averaged across 1000 simulations. Solid orange line: best fit obtained with a logistic sigmoid. For details, see text.

S3 Fig Two integrate-and-fire neurons n_1, n_2 connected by an excitatory synapse. Top panel: sinusoidal input current to n_1. Second panel: membrane potential and spikes of n_1. Third panel: membrane potential and spikes of n_1. Bottom panel: post-synaptic open probability $P_{n_1 \to n_2}$.

S4 Fig Comparison of integrate-and-fire neuron rates to rate neuron model predictions Top panel: rate of neuron n_1. Histogram computed from 1000 repetitions of the integrate-and-fire simulation. Lines show rate model predictions with a Poisson synapse, cf. Equation 9. Bottom panel: rate of neuron n_2.
