# Supplementary material for: A computational model for angular velocity integration in a locust heading circuit
Source: PLoS Comput Biol. 2024 Dec 20;20(12):e1012155. doi: 10.1371/journal.pcbi.1012155 (PMC11703117; doi:10.1371/journal.pcbi.1012155)
Supplement: S2 Text — (PDF) [file pcbi.1012155.s002.pdf]

S2 Text: Functional transmission delays between CL1a- and CL2-neuron populations

Turner-Evans et al. [1] used two fluorescent calcium sensors that radiate at different wavelengths to probe temporal relationships between the activities of P-EN- and E-PG-neurons of fruit flies walking in the dark. They found that P-EN activity in the EB leads E-PG activity. In their first experiment, they expressed the fast calcium sensors GCaMP6f [2] in P-EN-neurons, and jRGECO1a [3] in the E-PG population. Fig. 9G of [1] shows a positive angular difference between the compass bumps in P-EN and E-PG that increases with angular velocity, i.e. P-EN is ahead of E-PG. In a second experiment, the calcium sensors were switched between the neuron types (Fig. 9, supplement 2). A smaller, but still largely positive angular difference that increased with angular velocity was found in a small (N=5) number of walking flies.

Here, we tried to use their data for an order-of-magnitude estimation of the effective transmission delay Δt between P-EN- and E-PG-neurons. We based our estimation procedure on the following assumptions: first, the delay with which a calcium sensor responds to presynaptic spiking activity depends on the sensor, but not on the neuron on which it is expressed in. Second, calcium sensor expression does not alter the neuronal dynamics per se. We note that these assumptions may only be approximately correct, see e.g. [2,3]). Denote the time-dependent angles of the compass bump relative to a reference direction in the P-EN and E-PG population with $\phi_{P-EN}(t)$ and $\phi_{E-PG}(t)$, respectively. Assume that a fruit fly starts to rotate at orientation ϕ_0 with angular velocity ω. Let the unknown transmission delay between P-EN and E-PG be Δt. Then

$$\begin{aligned} \phi_{P-EN}(t) &= \omega \cdot t + \phi_0 && (1) \\ \phi_{E-PG}(t) &= \omega \cdot (t - \Delta t) + \phi_0 && (2) \end{aligned}$$

Both of these angles are unobservable. We also assume that the sensor delays Δt_r and Δt_g for jRGECO1a and GCaMP6f, respectively, are unknown. The data in [1] are based on the observable flurescence signals. In the first experiment, GCaMP6f is expressed in P-EN-neurons, and jRGECO1a is expressed in E-PG-neurons. Thus, the time courses of the angles corresponding to the peak amplitudes can be expressed as

$$\begin{aligned} \phi_{P-EN}(t)^g &= \omega \cdot (t - \Delta t_g) + \phi_0 && (3) \\ \phi_{E-PG}(t)^r &= \omega \cdot (t - \Delta t - \Delta t_r) + \phi_0 && (4) \\ \Rightarrow \phi_{P-EN}(t)^g - \phi_{E-PG}(t)^r &= \omega \cdot \underbrace{(\Delta t_r - \Delta t_g + \Delta t)}_{\alpha_{rg}} && (5) \end{aligned}$$

The slope α_{rg} of the relationship between the angular differences and the (known) angular velocity can therefore be estimated by a linear regression. For the opposite calcium sensor combination, we find

$$\begin{aligned} \phi_{P-EN}(t)^r &= \omega \cdot (t - \Delta t_r) + \phi_0 && (6) \\ \phi_{E-PG}(t)^g &= \omega \cdot (t - \Delta t - \Delta t_g) + \phi_0 && (7) \\ \Rightarrow \phi_{P-EN}(t)^r - \phi_{E-PG}(t)^g &= \omega \cdot \underbrace{(\Delta t_g - \Delta t_r + \Delta t)}_{\alpha_{gr}} && (8) \end{aligned}$$

The mean of the slopes therefore is the desired Δt. We estimate the slopes by Bayesian linear regression using the PyMC package [4] (version 5), which yields

$$\Delta t = \frac{\alpha_{gr} + \alpha_{rg}}{2} \approx 132\,\mathrm{ms} \pm 16\,\mathrm{ms} \tag{9}$$

This effective transmission delay is at least an order of magnitude larger than common membrane time constants, which justifies the use of a steady-state neuron model with (slower) dynamical synapses.

References

[1] Turner-Evans D, Wegener S, Rouault H, Franconville R, Wolff T, Seelig JD, et al. Angular velocity integration in a fly heading circuit. eLife. 2017;6:e23496. doi:https://doi.org/10.7554/eLife.23496.001.

[2] Chen T, Wardill T, Sun Y, Pulver S, Renninger S, Baohan A, et al. Ultrasensitive fluorescent proteins for imaging neuronal activity. Nature. 2013;499:295–300. doi:https://doi.org/10.1038/nature12354.

[3] Dana H, Mohar B, Sun Y, Narayan S, Gordus A, Hasseman JP, et al. Sensitive red protein calcium indicators for imaging neural activity. eLife. 2016;5:e12727. doi:http://dx.doi.org/10.7554/eLife.12727.

[4] Oriol AP, Virgile A, Colin C, Larry D, J FC, Maxim K, et al. PyMC: A modern and comprehensive probabilistic programming framework in python. PeerJ Computer Science. 2023;9:e1516. doi:https://doi.org/10.7717/peerj-cs.1516.
