# Supplementary figures and images for: A computational model for angular velocity integration in a locust heading circuit
Source: PLoS Comput Biol. 2024 Dec 20;20(12):e1012155. doi: 10.1371/journal.pcbi.1012155 (PMC11703117; doi:10.1371/journal.pcbi.1012155)

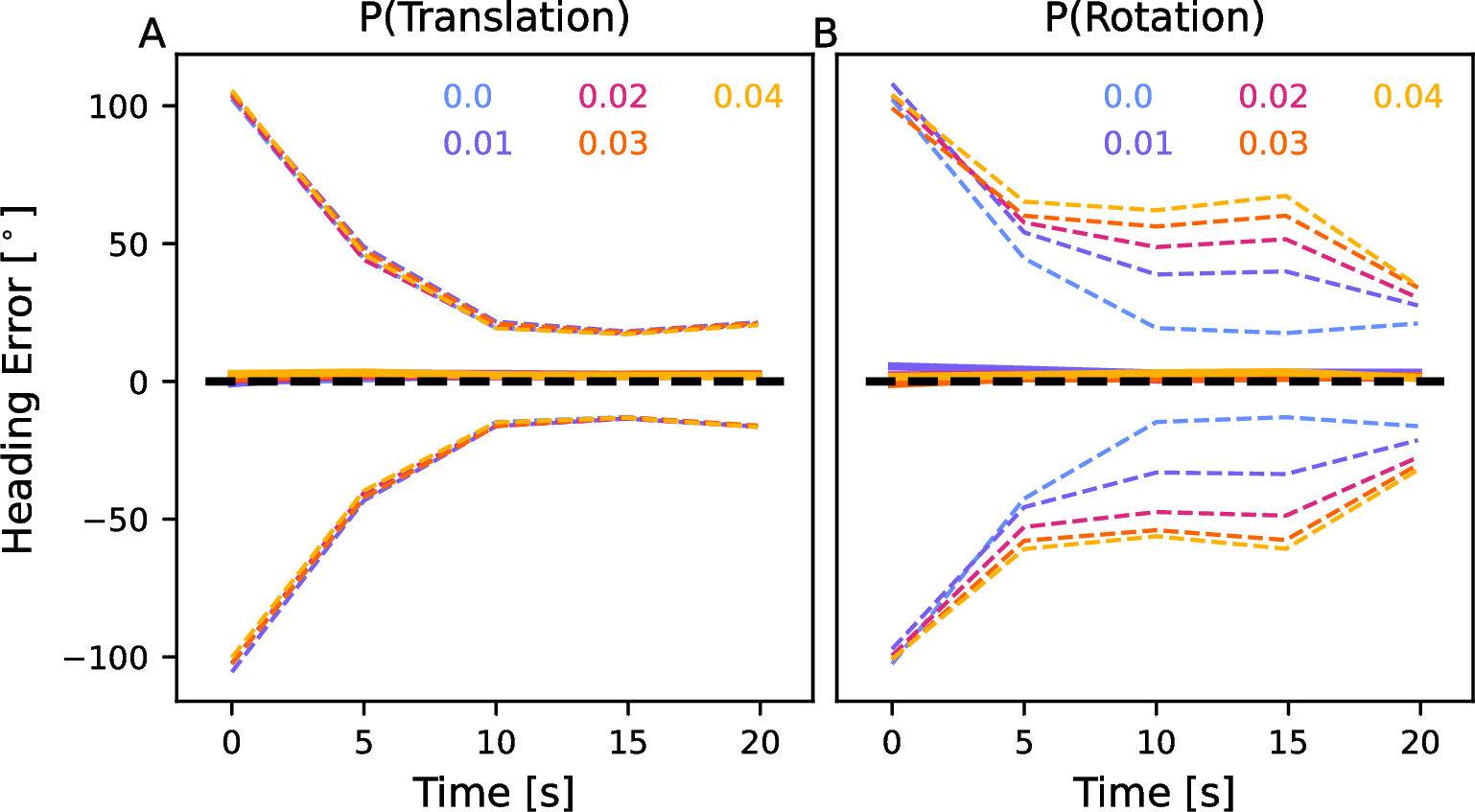

Supplement: S1 Fig — Mean-squared deviation between the agent’s heading estimate and the ground truth heading direction, averaged over 1000 trials lasting 20 s each, recorded every 5 s. Panels A and B demonstrate the ability of the agent to maintain straight-line orientation under conditions with different probabilities of being translated (A) or rotated (B) by wind. Solid lines show the mean angular deviation, dashed lines are mean ± one standard deviation. For details, see text. (TIF) [file pcbi.1012155.s003.tif]

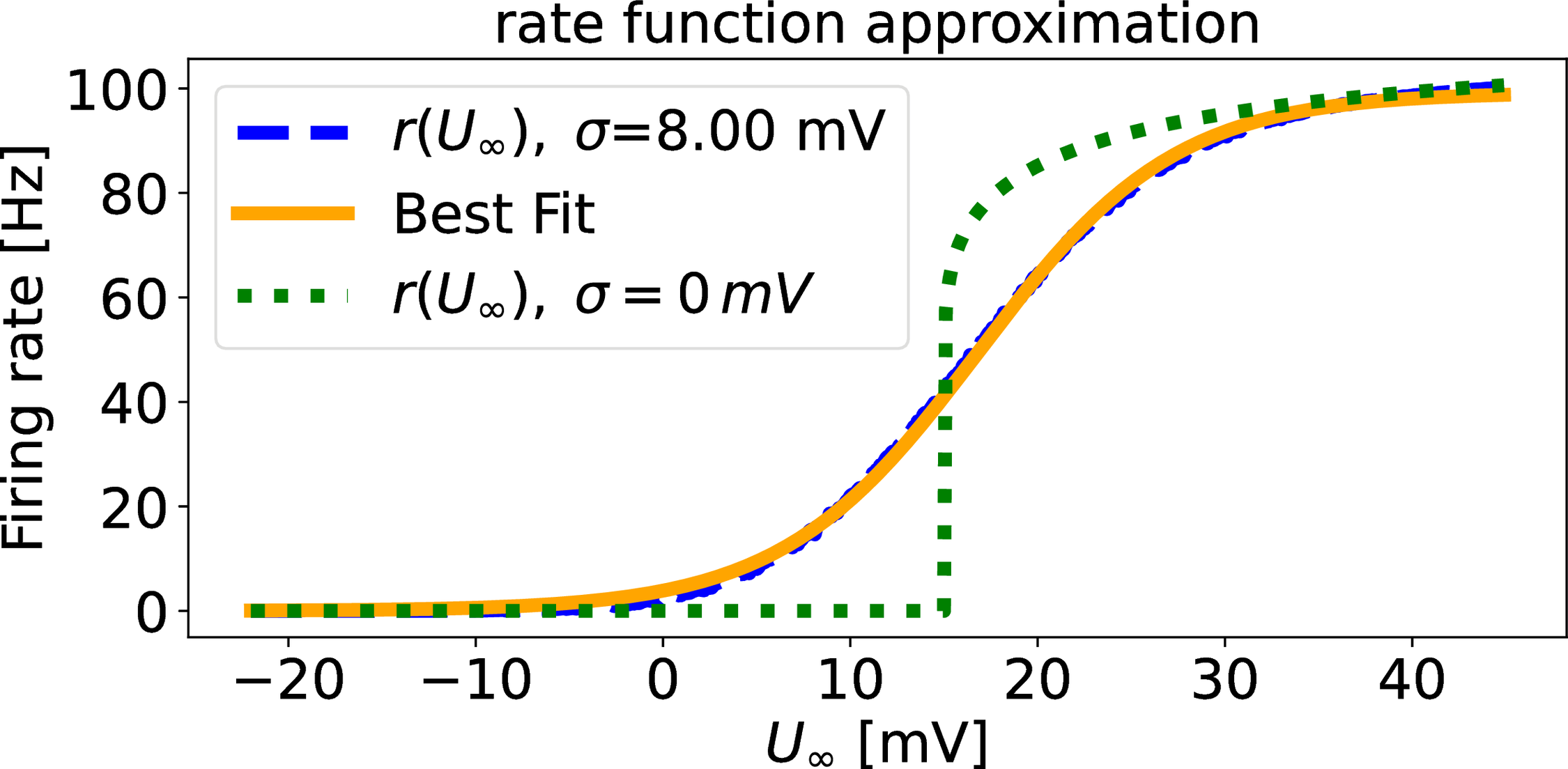

Supplement: S2 Fig — Green dotted line: rate function of the ideal integrate-and-fire neuron without noise. Blue dashed line: rate function with σ = 8 mV membrane noise, averaged across 1000 simulations. Solid orange line: best fit obtained with a logistic sigmoid. For details, see text. (TIF) [file pcbi.1012155.s004.tif]

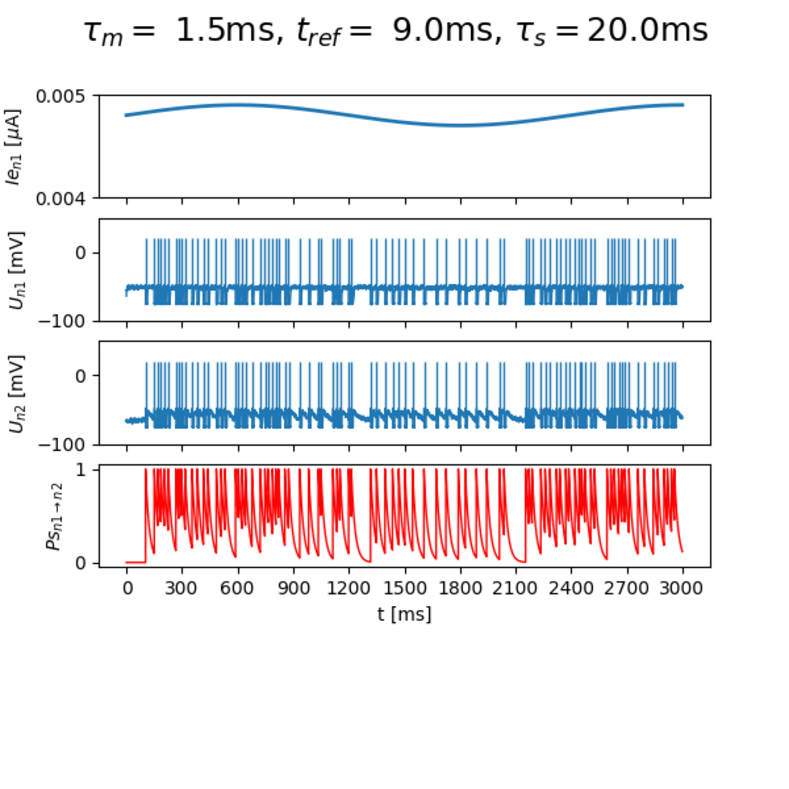

Supplement: S3 Fig — Top panel: sinusoidal input current to n1. Second panel: membrane potential and spikes of n1. Third panel: membrane potential and spikes of n1. Bottom panel: post-synaptic open probability Pn1→n2. (TIF) [file pcbi.1012155.s005.tif]

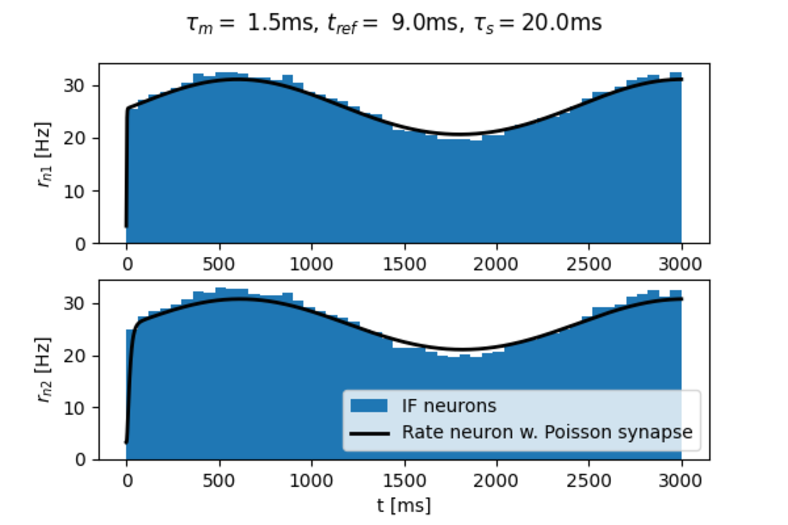

Supplement: S4 Fig — Top panel: rate of neuron n1. Histogram computed from 1000 repetitions of the integrate-and-fire simulation. Lines show rate model predictions with a Poisson synapse. Bottom panel: rate of neuron n2. (TIF) [file pcbi.1012155.s006.tif]
