# Supplementary material for: A computational model for angular velocity integration in a locust heading circuit
Source: PLoS Comput Biol. 2024 Dec 20;20(12):e1012155. doi: 10.1371/journal.pcbi.1012155 (PMC11703117; doi:10.1371/journal.pcbi.1012155)
Supplement: Attachment — Submitted filename: Pabst et al PLoS Comp Bio 2024.pdf [file pcbi.1012155.s007.pdf]

Review on the manuscript
"A computational model for angular velocity integration in a locust heading circuit"
by Pabst et al.

This is an exploratory study about angular velocity integration in locusts, based on an alternative mechanism compared to the fly literature, which makes it a very interesting piece of work. As a proof of principle, the manuscript is highly valuable and I support publication once the following points have been addressed.

Mayor points:
The authors claim to present "a novel model for angular velocity integration" (lines 15/16). This statement is formally correct if it refers to the specific application to the locust heading system but an exaggeration regarding the computational model itself, which was proposed almost 30 years ago (reference 62). I do not understand why this seminal paper is cited only a single time and suggest that the authors give proper credit to K Zhang.

Similarly, a more balanced view regarding the experimental evidence for the key ingredient of the model – multiplicative modulation – should be presented (lines 241-251): The existence of neuro-modulatory transmitters in tangential neurons is a necessary condition for the model to work but it is not sufficient, as this requires that the release of those transmitters can be up- and downregulated with the right time-course; long-lasting neuropeptides will not be able to do the job.

The mathematical framework of the paper rests on the assumption that synaptic time-constants (taken as tau_s= 30 ms on line 721 and 20 ms on line 736 (why these two different values?)) are much larger than membrane time constants (tau_m=1.5 ms), see, e.g., line 708. To support their parameter choice, the authors state that "tau_m = 1.5 ms is common in cortical neurons" (lines 129 and 704) with reference to Dayan and Abbott (2001). However, Dayan and Abbott rather state (Chapter 5): "The membrane time constant sets the basic time scale for changes in the membrane potential and typically falls in the range between 10 and 100 ms." Similarly, I cannot find a justification for tau_s=30 ms in Dayan and Abbott (2001) either, contrary to what Pabst et al. write (line 721/722). So it is not clear to me whether the presumed separation of time scales actually holds.

The absolute refractory period tau_ref is assumed to be 9 ms (line 721), i.e., the typical inter-spike interval is just a bit more than twice as long. This raises the question whether the Poisson assumption implicit in equations (4) and (19) is justified. A numerical investigation of the full equations (including refractoriness) is needed to substantiate the authors' heuristic argument. Note in passing that r_i in both equations should be marked as time-dependent.

I do not understand the term "steady-state firing rate neurons" as it suggests that the firing rate of those model neurons does not change in time. However, this would make any time-dependent operation of the system impossible and is also at odds with other parts of the manuscript. For example, equation (19) describes the time-evolution of P_{s,i} which causes U_infty to vary in time (equation 13) and this implies U to be a dynamic variable (equation 14), which seems to contradict the term "steady-state firing rate neurons". A different term might solve these problems.

The regularization used by Pabst et al. "promoted similar values for all synaptic weights across repeated connectivity structures" (line 299/300). Doesn't this severely limit any comparisons between the model architecture and real connectivity structures as other regularizations are likely to result in different model architectures?

Finally, the authors may also want to reconsider their nomenclature. Adopting the drosophila terms, as in the review by K Pfeiffer (Current Opinion Insect Science, 2023) might help to reach a larger audience as the similarities and differences between the two species are much easier accessible when matching terms are used.

Minor points:
line 144, equation (3): the term "99.6 Hz" suggests a precision for the parameters of the logistic fit that is not warranted by the procedure used. Why not simply assume "100 Hz"? Similar arguments apply to the two parameters in the denominator.

line 146, "inhomogeneous Poisson process" implies a time-dependent rate, so r_i (in the same line) should have a time-argument.

line 160: "angular speed neurons": Misleading term, as these neurons do *not* encode speed (|v|) but rather rectified velocities (max(0,v) and max(0,-v), respectively) .

line 333: The model performance was calculated based on the true and estimated heading directions after some fixed time period. However, a small mismatch at the end of the experiment does not imply a small mismatch throughout the entire time course – the system might have drifted by 360°. I don't think that this happens (often), but one nevertheless has to check the possibility of such an error.

line 351ff – agent simulation: I miss the characterization of the path to be followed (straight or stochastic changes of velocity or direction, etc).

line 496: I don't understand the statement that a "weight noise up to 5% only marginally increased the number of larger errors in the heading estimate" – for a 5% noise level, the heading distribution is essentially flat (see Fig 5C)!

line 531: Figure S5A-B has a poor color code and one cannot really see what the authors want to show. In addition, I could not find information on the rather large temporal discretization (5s).

line 532: the statement "without (A) and with (B) wind-induced perturbations" does not fit with what is shown in the figure.

line 532/533: The statement "These results demonstrate the simulated agent's capability to carry out goal-directed locomotion in dynamic environments..." seems to need an explanation as the angular mismatch is typically at least 10° (7C, 7D).

line 534ff (Caption of Fig 7): Why is the distribution of angular deviations bimodal?

line 534ff (Caption of Fig 7): How many time steps do 12.5s correspond to?

line 687: "Panels A and B demonstrate the impact of the probability of a wind gust..." Shouldn't it read "Panels A and B demonstrate the impact of a wind gust with a certain the probability ..."? (a wind gust might have an impact but not its probability)

line 715, equation (15): Shouldn't the "log" be a "ln"?

lines 717 and 721: V_{reset} should be U_{res}

line 719/720: "deviations whose average effect can be well described by a logistic sigmoid" a reference for this statement would be appreciated.

line 721/722: The authors list parameter values "which are typical values for cortical neurons" and cite the textbook by Dayan and Abbott (2001) to support this statement. I could not find these parameter values in Dayan and Abbott (2001).

line 723: I do not understand the argument leading to the assumption that thermally induced voltage fluctuations are around 8mV. Given the authors' own argument, they should rather be around 25mV (which clearly shows that the authors' logic does not hold). In this context, reference 84 appears to be of little help as it deals with an in-vivo preparation where synaptic inputs might be the main cause of the observed voltage fluctuations. Finally, theoretical work by Steinmetz et al. (J Comp Neurosci 2000) shows that thermal fluctuations are typically well below 2mV.

line 727: Average rate functions might have a sigmoid shape. This does, however, not provide any justification for the specific parameter values.

line 754ff: I may miss a crucial point, but I simply do not understand how a synapse with fixed reversal potential can be both, excitatory and inhibitory. In addition, I would like to see a specific reference for the statement that 65mV "is approximately the reversal potential for cations relative to the resting potential. Finally, equation 22 does not seem to be compatible with equation 13.

line 764ff: "... P-EN activity precedes P-EN activity in the EB." There is something wrong or missing.

line 781: "... transmission delay between E-PG and P-EN ..." Shouldn't it be the other way round?

line 785: one "P-EN" should be an "E-PG".

line 792: Why does a long transmission delay justify "the use of a steady-state neuron model"? Typically, delay-differential equations with large negative feedback show oscillations.

Figure 7A: Why does the agent not improve its heading direction with increasing time? Shouldn't it be able to compare its heading direction with the goal direction? And why do the added perturbations in 7B improve the situation?
