# Supplementary material for: A computational model for angular velocity integration in a locust heading circuit
Source: PLoS Comput Biol. 2024 Dec 20;20(12):e1012155. doi: 10.1371/journal.pcbi.1012155 (PMC11703117; doi:10.1371/journal.pcbi.1012155)
Supplement: Attachment — Submitted filename: Pabst_2024_review.pdf [file pcbi.1012155.s008.pdf]

The manuscript by Pabst et al. puts forward a computational model for angular velocity integration in the central complex of the locust. The proposed model is noteworthy in that it differs significantly from previous models grounded in experimental data from *Drosophila*. Whereas experiments in flies indicate that each side of the protocerebral bridge represents the full 360º of azimuthal space, the proposed model assumes that each side of the bridge represents only 180º based on intracellular recordings performed in the locust. In my view, the present study is of value because the hypothesis that the protocerebral bridge of the locust only represents 360º is intriguing—computational studies that investigate the plausibility of this hypothesis (or lack thereof) could provide concrete predictions for experimentally testing this model or prompt a re-evaluation of the data that originally inspired it.

Overall, I found that the manuscript would greatly benefit from improvements in clarity, in particular with regards to providing the reader with an intuition for how the model works. I also think that the biological plausibility of the proposed model, as well as experiments that could test it, should be discussed more.

<u>Major points</u>

1. It is not clear how the dynamics in CL1a/CL2 connectivity actually cause the bump of activity in the bridge to be updated during turns. In the *Drosophila* heading system, the imbalance in activity between left and right PEN neurons and the anatomical shift from the bridge to the ellipsoid body are two key features for updating the position of the EPG bump of activity. From my understanding, neither of the features play a role in updating the position of the EPG/CL1a bump of activity in the proposed model. Instead, the authors posit the existence of some neuron that modifies the synaptic weights of the CL1a and CL2 neurons via some neuromodulator depending on the insect's angular velocity. Presumably differences in the activity of CL2 neurons somehow cause the CL1a bump to move, but how exactly was not clear from Figure 4 (I did not find the force-directed graphs useful). The authors should explain this more clearly. Perhaps a cartoon highlighting the key connections that are important for updating the CL1a bump position during turns and how they are dynamical modified would be helpful.
2. On line 584 the authors highlight that it is unclear whether a 360º model of the protocerebral bridge is compatible with recurrent inputs from TB1/2 neurons (Delta7 neurons in flies). This is a very good point. The fact that individual TB1/2 neurons have outputs in the bridge every 8 glomeruli would seem to imply that the preferred heading angles should be similar, or the same, every 8 glomeruli, which is inconsistent with the 360º model of the bridge. I think this should be addressed in the present paper by incorporating these connections in the proposed model.
3. The authors should propose experiments that could be performed by future studies to rigorously test their model. For example, does their model make any predictions about the functional properties of CL2 and CL1a neurons that are not expected based on the *Drosophila* heading system?

Minor points

1. It would be helpful if Figure 1 also included the expected activity pattern in the CBL/EB since this a significant difference between the fly heading system and the proposed model. It seems likely that the absence of a coherent bump of activity in the CBL (i.e., the presence of two intercalated 180º representations) might pose additional challenges to the system.
2. Is it necessary for both the CL1a and CL2 synapses to be modulated by angular velocity for the model to work? Why did the authors choose to do this?
3. On line 367 it says that in the model by Stone et al. 2017 *"the current heading direction is represented by two compass bumps across 16 CL1a-neurons. This is transformed into a single bump across 8 TB1-neurons."* This is confusing because the spatial pattern activity of TB1 neurons in the bridge consists of two bumps of activity in Stone et al. 2017 (see Figure 5h).
4. On line 455 the authors state that *"the synaptic strengths are up- and down-regulated during turns, but the polarity of synapses (excitatory or inhibitory) is not altered by neuromodulation"*. However, the sign of the weights of several neurons in Figure 4B change in Figure 4C. Are these not synaptic weights? Please clarify.
5. In the proposed model, not only do the connections between a CL2 neuron and a CL1a neuron get up or down-regulated depending on whether the insect is turning clockwise or counter-clockwise, but also different CL2-to-CL1a connections (from the same pre-synaptic CL2 neuron) are modulated differently during a same turn. I believe these two features of the model requires two types of neuromodulators *and* differences in how these two neuromodulators are received. This was not clear in the Neurobiological plausibility section (which made it seem like only one of these features would be necessary).
6. In *Drosophila*, the GLNO neurons provide rotational velocity inputs to PEN neurons (Hulse et al. 2023 https://doi.org/10.1101/2023.09.25.559373). Are the corresponding neurons in locusts (the TNL neurons) also tuned to optic flow? If so, would these inputs be compatible with the proposed 360º model of the bridge?
7. In the proposed model, does the bump of activity drift when the angular velocity is zero? This is somethings that was investigated by Noorman et al. 2022 (https://doi.org/10.1101/2022.05.23.493052).
