# Supplementary material for: A computational model for angular velocity integration in a locust heading circuit
Source: PLoS Comput Biol. 2024 Dec 20;20(12):e1012155. doi: 10.1371/journal.pcbi.1012155 (PMC11703117; doi:10.1371/journal.pcbi.1012155)
Supplement: Attachment — Submitted filename: response_letter.pdf [file pcbi.1012155.s009.pdf]

We thank both reviewers very much for for their thoughtful and constructive reviews. We truly appreciate the insightful comments and have addressed them in detail below. All line numbers refer to the version of the manuscript with highlighted changes.

1 Review A

This is an exploratory study about angular velocity integration in locusts, based on an alternative mechanism compared to the fly literature, which makes it a very interesting piece of work. As a proof of principle, the manuscript is highly valuable and I support publication once the following points have been addressed.

1.1 Major points

1. The authors claim to present "a novel model for angular velocity integration" (lines 15/16). This statement is formally correct if it refers to the specific application to the locust heading system but an exaggeration regarding the computational model itself, which was proposed almost 30 years ago (reference 62). I do not understand why this seminal paper is cited only a single time and suggest that the authors give proper credit to K Zhang.

   We have clarified that the novelty of our model lies in its specific application to an insect heading circuit in the abstract: "We present a model for angular velocity integration in a desert locust heading circuit, applying concepts from early theoretical work on heading circuits in mammals to a novel biological context in insects" (lines 16-20).
   We have further mentioned Zhang's work in more detail in the "Introduction" section: "A model of angular velocity integration in a fruit fly heading circuit (Turner-Evans et al., 2017) includes two types of columnar neurons, E-PG-neurons encoding heading and P-EN-neurons conjunctively encoding heading and angular velocity. Within this model, asymmetric activation of P-EN-neurons in the two halves of the PB occurs based on the fruit fly's turning direction, resulting in a shift of E-PG and P-EN activity maxima in the EB and the PB through the circuit's connectivity. This mechanism is consistent with an early theoretical framework of self-motion integration in a vertebrate heading circuit (Zhang, 1996).This framework proposed additive and multiplicative modulation that introduces asymmetries to the circuit connectivity as two alternatives to shift the compass bump. The model by Turner-Evans et al. (2017) fits the additive modulation mechanism described by Zhang (1996)" (lines 95-105).
   And further in the "Introduction" section we have added: "In previous

work, using a simplified model with linear neural units, discrete-time updates and binary rotation encoding (left vs. right), we showed that the observed heading encoding in the locust could in principle be shifted appropriately by a multiplicative rotation-dependent modulation of the firing rate (Pabst et al., 2022; Zittrell et al., 2023). Unlike the additive modulation mechanism observed in the fruit fly, our locust model aligns with the multiplicative modulation introduced by Zhang (1996) and suggests a potential biological implementation in an insect heading circuit." (lines 116-122).

Lastly, we have expanded the "Discussion" section to mention alternative biological implementations of multiplicative modulation suggested by Issa and Zhang (2012): "While our model focuses on synaptic-level neuromodulation, other mechanisms could also achieve multiplicative effects. For instance, multiplication can occur through exponentiation by active membrane conductances of summed logarithmic inputs, nonlinear dendritic interactions, or network dynamics (Issa & Zhang, 2012)." (lines 654-658).

2. Similarly, a more balanced view regarding the experimental evidence for the key ingredient of the model – multiplicative modulation – should be presented (lines 241-251): The existence of neuro-modulatory transmitters in tangential neurons is a necessary condition for the model to work but it is not sufficient, as this requires that the release of those transmitters can be up- and downregulated with the right time- course; long-lasting neuropeptides will not be able to do the job.

   We have added the following statement to the "Neurobiological plausibility" subsection of the "Discussion" section to emphasize this requirement: "While the presence of neuromodulatory transmitters suggests potential for modulation, their effectiveness in our model relies on more than just their presence. Specifically, for the model to work as intended, these transmitters must be able to both up- and down-regulate synaptic strengths with appropriate timing. Long-lasting neuropeptides, for instance, may not be able to provide the rapid and reversible modulation required. Future experiments should focus on determining whether the temporal dynamics of neurotransmitter release and modulation align with the requirements of our model" (lines 735-742).

3. The mathematical framework of the paper rests on the assumption that synaptic time-constants (taken as $\tau_s = 30\,\text{ms}$ on line 721 and $20\,\text{ms}$ on line 736 (why these two different values?)) are much larger than membrane time constants ($\tau_m = 1.5\,\text{ms}$), see, e.g., line 708. To support their parameter choice, the authors state that "$\tau_m = 1.5\,\text{ms}$ is common in cortical neurons" (lines 129 and 704) with reference to Dayan and Abbott (2001). However, Dayan and Abbott rather state (Chapter 5): "The membrane time constant sets the basic time scale for changes in the membrane potential and typically falls in the range between $10\,\text{ms}$ and $100\,\text{ms}$."

   Our choice of τ_m: in contrast to the integrate-and-fire model in Dayan

& Abbott, we model the refractory period explicitly with $\tau_{ref} = 9\,\text{ms}$. Combined with a membrane time constant $\tau_m = 1.5\,\text{ms}$, our model has an effective refractory period of $\approx 10\,\text{ms}$. The short τ_m results from the membrane parameters of a Hodgkin-Huxley model at rest: the residual opening of the potassium and sodium channels leads to an increase of the membrane conductance, thus reducing τ_m to ca. 1.5 ms. This value was computed by a Hodgkin-Huxley simulation without driving input, using the parameters on page 173 in Dayan & Abbott. We have adjusted the text in the subsection "Neuron model" in the section "Model and methods" accordingly (lines 148-152): "Estimates for τ_m vary with neuron type. While $\tau_m = 1.5\,\text{ms}$ can be derived from the resting state conductance of a Hodgkin-Huxley model with the parameters values given in Dayan & Abbott (2001), the membrane time constants used in the fruit fly CX model of Turner Evans et al. (2017) are larger by an order of magnitude to capture the observed delays between E-PG and P-EN activity in walking flies (Turner Evans et al., 2017)."

Effects of τ_m choice on rate function: To illustrate that this choice does not have a large effect on the model, we simulated average rate functions for different choices of τ_{ref}, τ_m subject to the constraint $\tau_{ref} + \tau_m = 10.5ms$. The script `invertpy/brain/centralcomplex/cl1acl2/fit_rate_function.py` runs these simulations and computes the best fitting sigmoids:

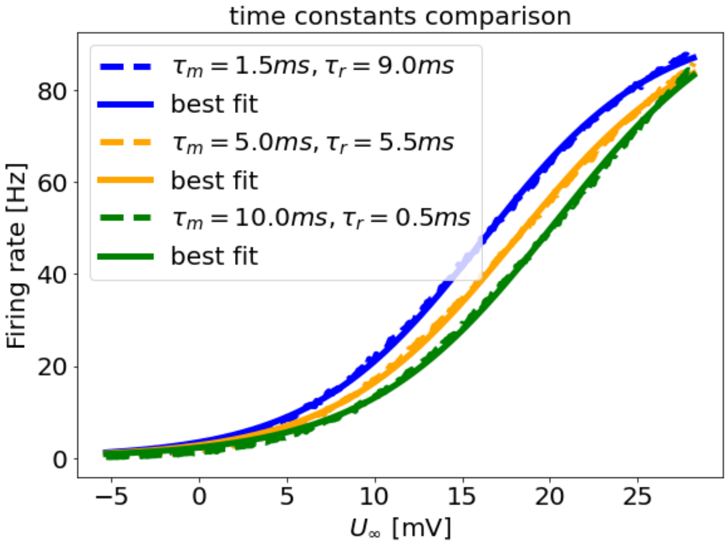

Especially in the relevant range of 20 Hz and 30 Hz, the differences in the rate functions resulting from trading off the two time constants amount to a horizontal shift, which can be compensated by matching bias choices in our machine learning approach. For larger τ_m, reaching the observed

firing rates of $\approx$ 20 Hz and 30 Hz becomes increasingly more difficult for U_∞ in a physiologically plausible range:

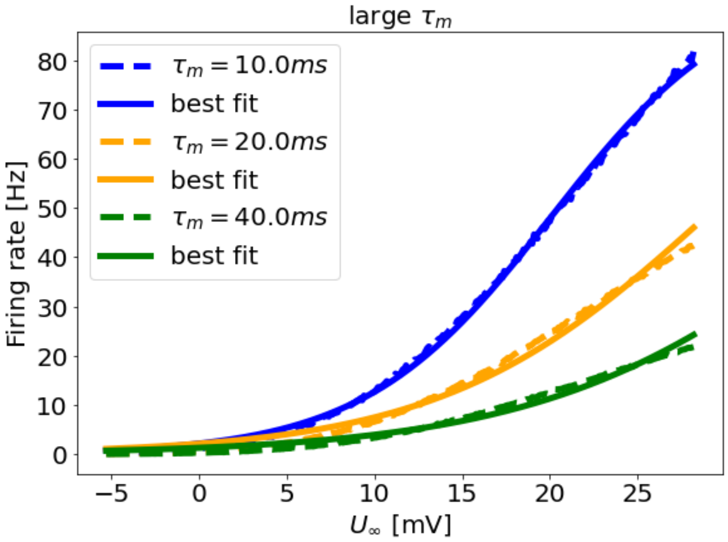

Effects of τ_m choice on integration performance: to demonstrate that it is possible to find synaptic weights which yield good integration performance for different membrane model parameters, we re-trained the network for different choices of τ_m and τ_{ref} and repeated the integration simulation (as shown in Figure 5 in the manuscript) with the same noise parameters for $\tau_s = 20ms, \tau_{s.mod} = 30ms$):

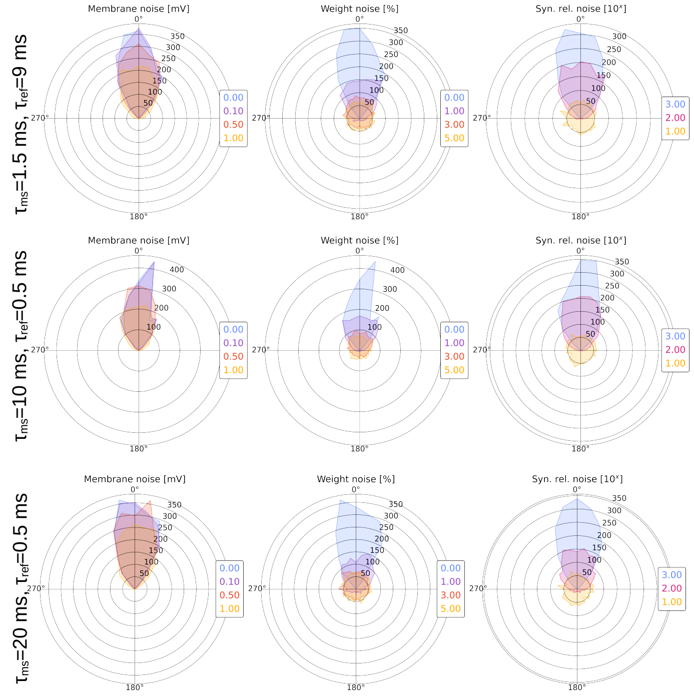

The top row shows simulations with the same membrane parameters as in the manuscript. In the middle row, we used a very short explicit refractory period and a longer $\tau_m = 10\,\text{ms}$ (cf. green curves in the 'time constants comparison' plot above). In the bottom row, we used an even longer $\tau_m = 20\,\text{ms}$ (cf. yellow curve in the 'large τ_m' plot above). The integration performance, especially also under noisy conditions, is comparable for all choices of membrane parameters. Hence, we propose to keep our modeling choice of $\tau_m = 1.5\,\text{ms}$ and $\tau_{ref} = 9\,\text{ms}$

4. Similarly, I cannot find a justification for $\tau_s = 30\,\text{ms}$ in Dayan and Abbott (2001) either, contrary to what Pabst et al. write (line 721/722). So it is not clear to me whether the presumed separation of time scales actually holds.

   Thank you for catching this mistake, the value we used was $\tau_s = 20\,\text{ms}$, and typos throughout the manuscript have been corrected.

   We have changed the text to: "Precise values for τ_s in the locust are currently unknown, but drawing on our estimates from available fruit fly data, we argue for moderately slow synapses (see section "Functional transmission delays between CL1a- and CL2-neuron populations"). We chose $\tau_s = 20\,\text{ms}$. This choice provides a balance between two important

factors. First, it helps smooth out noise in angular velocity integration by acting as a low-pass filter. Second, it allows the compass representation to respond effectively to angular velocity inputs, within the range we have modeled: $[-150\,°/\mathrm{s}, 150\,°/\mathrm{s}]$" (lines 948-954).

The justification for using a longer $\tau_s = 20\,\mathrm{ms}$ or more is not given in Dayan and Abbott (2001) but results from our analysis of the functional transmission delays between the P-EN- and E-PG-neurons of the fruit fly in the supporting information of the manuscript. A long τ_s can explain spiking activity in a post-synaptic neuron after pre-synaptic activity has ceased. This phenomenon, which was observed in the fruit fly (cf. supporting information on functional transmission delays), cannot be accounted for by long membrane time constants, due to the hyperpolarization after a spike, which will suppress further spikes after the input signal has ceased. However, the precise value of the synaptic time constants in the locust is not known at the moment. To show that a range of synaptic time constants will lead to good integration performance, we re-trained the network with different time constants and performed the same integration simulations as in the manuscript:

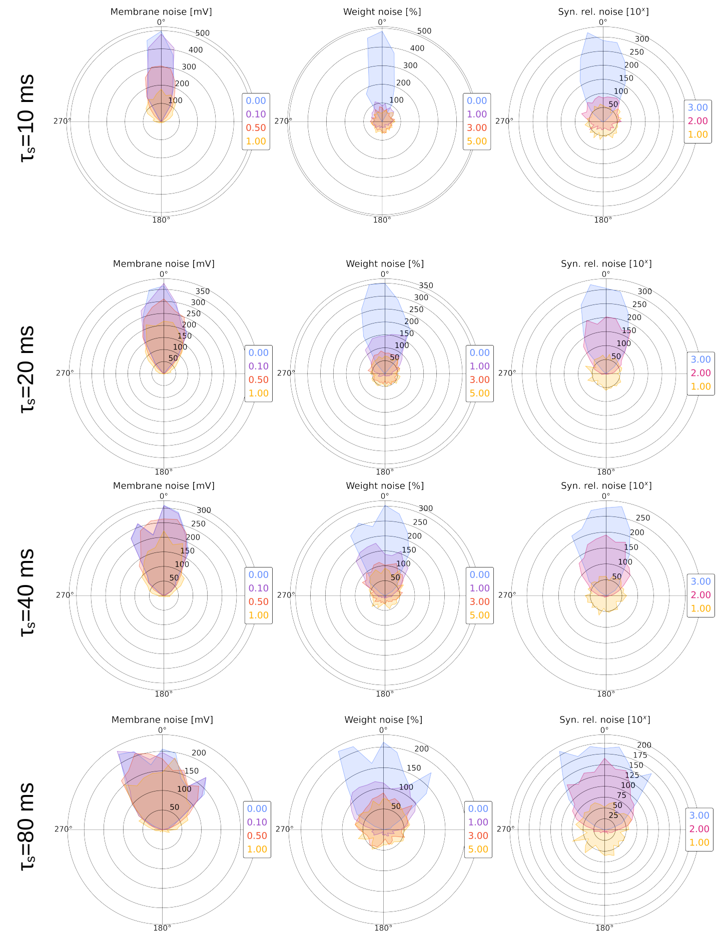

Integration simulation with synaptic time constants $\tau_s \in \{10\,\mathrm{ms}, 20\,\mathrm{ms}, 40\,\mathrm{ms}, 80\,\mathrm{ms}\}$ and $\tau_{s,mod} = \tau_s + 10\,\mathrm{ms}$ like in the manuscript. Shorter τ_s yield better integration under noise-free conditions. However, longer τ_s yield more stable integration performance under noisy conditions, particularly synaptic release noise. Our choice $\tau_s = 20\,\mathrm{ms}$ is a good compromise across all noise levels. Further experimental research is needed to determine physiologically correct values.

5. The absolute refractory period $\mathrm{tau}_{\mathrm{ref}}$ is assumed to be $9\,\mathrm{ms}$ (line 721), i.e., the typical inter-spike interval is just a bit more than twice as long. This raises the question whether the Poisson assumption implicit in Equations (4) and (19) is justified. A numerical investigation of the full equations

(including refractoriness) is needed to substantiate the authors' heuristic argument. Note in passing that $\mathrm{r_i}$ in both equations should be marked as time-dependent.

We now mark $\mathrm{r_i(t)}$ explicitly as time dependent in Equations (4) and (19), as suggested.

Validity of Poisson assumption: to demonstrate that the Poisson assumption is a feasible approximation in our setting, we simulated the expected post-synaptic channel opening probability P(open) assuming a spike-generating process with gamma-distributed inter-spike-intervals. We used a pre-synaptic rate with sinusoidal modulation, such as our model CL1a- or CL2-neurons would receive from their pre-synaptic partners. We varied the shape parameter of the gamma distributions to investigate the effects of different refractory periods $T_r \in \{5\,\mathrm{ms}, 10\,\mathrm{ms}, 20\,\mathrm{ms}\}$, while keeping the effective pre-synaptic rate identical. Here, T_r is the most probable inter-spike-interval, not the absolute refractory period τ_{ref}.

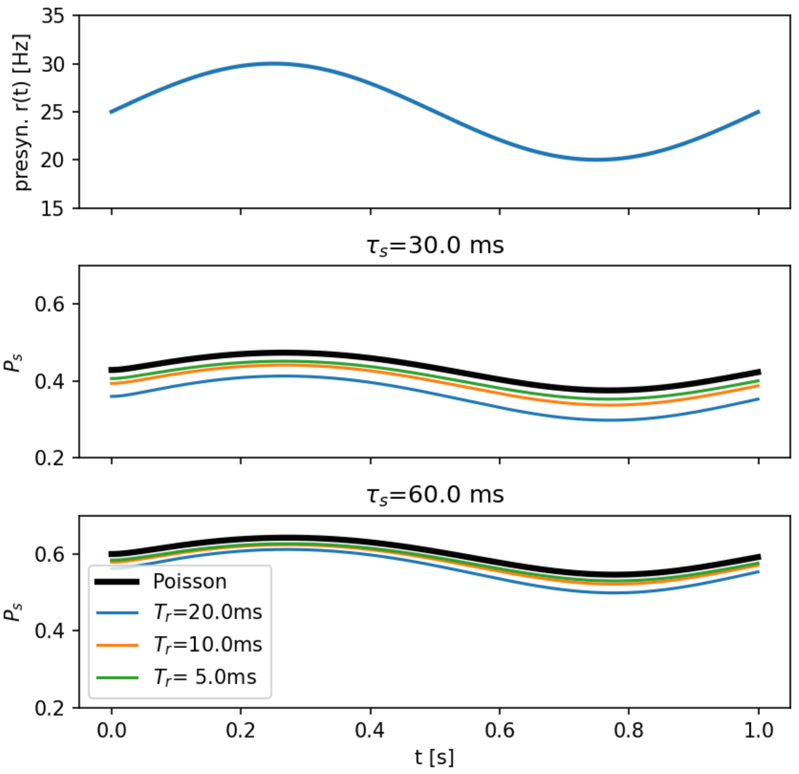

Even for the longest $T_r = 20\,\text{ms}$ (blue curve), only very modest changes in P(open) are resulting. Hence, the Poisson assumption does not invalidate the model. Also, a slightly reduced P(open), such as for $T_r = 20\,\text{ms}$ could be compensated by an increased synaptic conductance.

Comparison of the integrate-and-fire model with refractoriness to rate model with Poisson synapses: The following information has been added to the supplementary information section "Neuron model derivation", subsection "Synapse model" (lines 944-986): to show that our rate model is a good approximation to a spiking neuron, we connected two integrate-and-fire neurons with an excitatory synapse, using $\tau_s = 20\,\text{ms}$. We simulated neurons with $\tau_m = 1.5\,\text{ms}$ and $t_{ref} = 9\,\text{ms}$ as in the manuscript, and a relative synaptic conductance $\tilde{g}_{tot} = 0.5$ We simulated membrane noise with a standard deviation of 8 mV over a 20 ms time period. The first neuron was driven by a sinusoidal input current with a frequency of $150\frac{\text{deg}}{s}$ and a magnitude chosen to cause a rate variation similar to that in our model with the fastest angular velocity input. We plotted an exemplary trace of the input current Ie_{n1}, the spikes and membrane potentials U of the two neurons n_1 and n_2, and the post-synaptic open probability $P_{n_1 \to n_2}$:

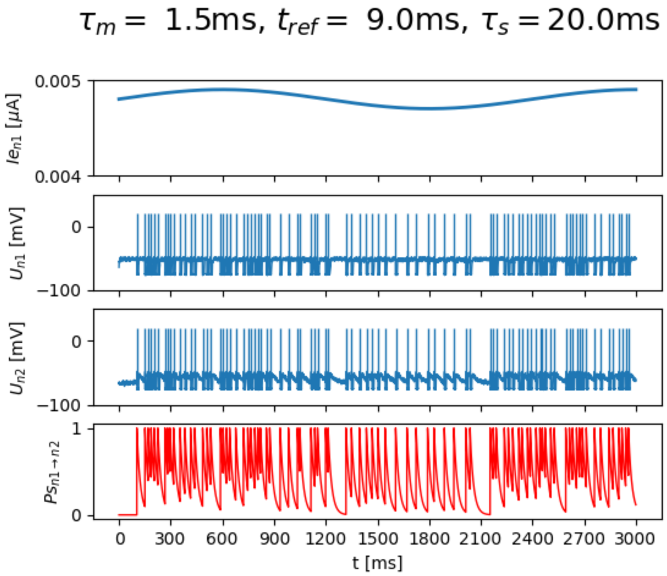

We repeated this simulation 1000 times, computed spike rate histograms

across simulations and evaluated the corresponding rate model with a Poisson synapse, using the sigmoid activation function from the manuscript.

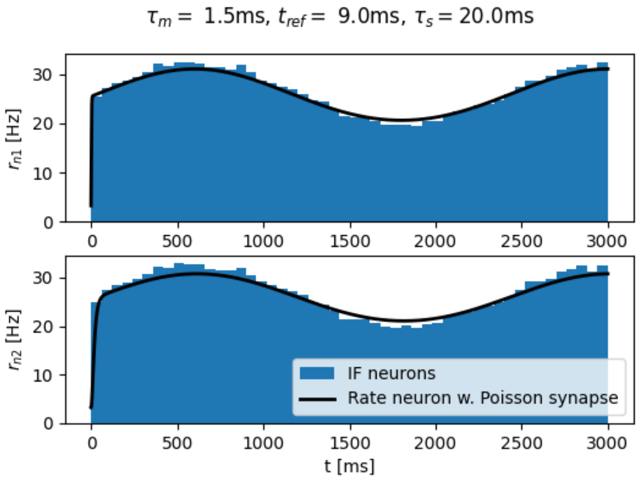

The top panel shows the rate of neuron n_1, the bottom panel neuron n_2. A good match between the two rate predictions can be observed. To achieve this match, we had to set the the Poisson synapse's $\tilde{g}_{tot} = 0.69$, i.e. slightly larger than in the IF model. This underscores our heuristic argument that the Poisson assumption does not invalidate our model in the presence of refractoriness.

Separation of timescales: even if our assumed separation of timescales would not hold, our Poisson rate model could still be a good approximation to an integrate-and-fire model with a suitable adaptation of parameters. To illustrate that, we repeated the comparison with $\tau_m = 10ms$, $\tau_s = 20ms$, i.e. comparable timescales between membrane and synapse. The rate function was adapted to these settings as described above under 'Effects of τ_m choice on rate function'.

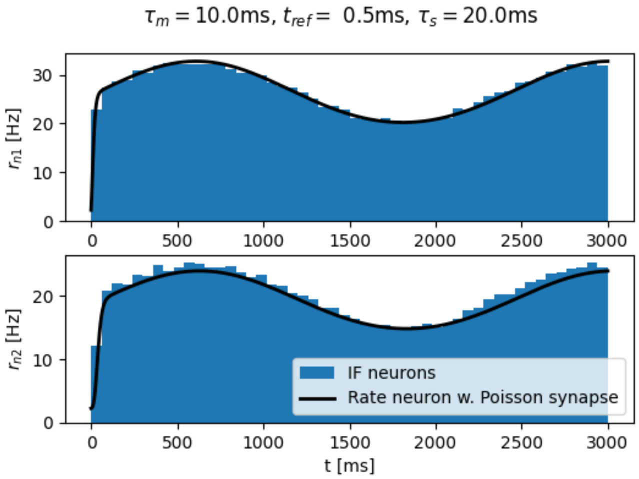

Here, too, a good match between the integrate-and-fire model and the Poisson rate model can be achieved. We conclude that a strict separation of timescales is not necessary for our approximation to hold. These simulations can be reproduced by running the script `invertpy/brain/centralcomplex/cl1acl2/IFNeuron.py` in the repository.

6. I do not understand the term "steady-state firing rate neurons" as it suggests that the firing rate of those model neurons does not change in time. However, this would make any time-dependent operation of the system impossible and is also at odds with other parts of the manuscript. For example, Equation (19) describes the time- evolution of $P_{s,i}$ which causes U_{infty} to vary in time (Equation (13)) and this implies U to be a dynamic variable (Equation (14)), which seems to contradict the term "steady-state firing rate neurons". A different term might solve these problems.

   In our model, the term "steady-state firing rate neurons" is used to describe neurons that achieve a stable firing rate after the system has settled from initial transients. This is due to the slow time constants of the synapses relative to the membrane time constant.

   We have added the following clarification in the "Neuron model" subsection in the "Model and methods" section: "In our computational model, "steady-state firing rate neurons" refers to a simplified representation where neurons are considered to achieve a stable firing rate after initial transients have settled. This stable firing rate is reached due to the slow dynamics of the synaptic inputs relative to the membrane time constant. Although the firing rate becomes stable after some time, it is still responsive to ongoing inputs and can change depending on the input dynamics. The steady-state potential U_∞ of the membrane, which evolves on the same slow time scale as the neuron's inputs, is [...]" (lines 156-163).

7. The regularization used by Pabst et al. "promoted similar values for all synaptic weights across repeated connectivity structures" (line 299/300). Doesn't this severely limit any comparisons between the model architecture and real connectivity structures as other regularizations are likely to result in different model architectures?

   Different forms of regularization could indeed restrict the exploration of certain feasible solutions. We have added the following paragraph to the Discussion section: "First, it was regularized by a simple yet reasonable assumption in the absence of detailed biological data: anatomically similar structures might perform similar physiological functions. This allowed us to investigate the robustness of physiological patterns across repeated structures without preemptively biasing the model towards more specific assumptions" (lines 711-715). We agree that future research will be essential in determining whether this assumption holds true. One of the strengths of our machine learning approach is its adaptability: new constraints, informed by emerging data, can easily be integrated into the regularization process. Retraining the model under these conditions will allow us to further refine its architecture in light of new biological insights.

8. Finally, the authors may also want to reconsider their nomenclature. Adopting the drosophila terms, as in the review by K Pfeiffer (Current Opinion Insect Science, 2023) might help to reach a larger audience as the similarities and differences between the two species are much easier accessible when matching terms are used.

   While we agree that aligning with the *Drosophila melanogaster* nomenclature could facilitate broader accessibility, we have chosen to retain the locust-specific nomenclature for the following reasons:

   1. **Historical precedence**: The locust nomenclature was established earlier, and the *Drosophila* terminology developed independently without referencing pre-existing names.
   2. **Unproven homologies**: The proposed correspondences between locust and *Drosophila* neuronal cell types remain unproven, with notable differences in morphology (e.g., TB1 vs. Delta7 neurons and also in the arborization domains of CL1a/E-PG and CL2/P-EN neurons).
   3. **Consistency**: Retaining the locust terminology maintains consistency with the existing locust literature, which is essential for cross-referencing earlier studies.
   4. **Cross-species comparison**: We have ensured that readers familiar with *Drosophila* terminology can follow the manuscript by providing corresponding terms in the text and a comparison table (Table 1).

   The increasing availability of comparative data suggests that *Drosophila* is, in many cases, a highly specialized insect, and findings from this species

cannot always be generalized to other insects. Our study may provide a valuable example of this, highlighting the need to preserve species-specific terminology where necessary.

1.2 Minor points

1. line 144, Equation (3): the term "99.6 Hz" suggests a precision for the parameters of the logistic fit that is not warranted by the procedure used. Why not simply assume "100 Hz"? Similar arguments apply to the two parameters in the denominator.

   The values in Equation (3) are a direct result of the specific parameters used in our simulations, and we retained this precision to reflect the exact configuration employed in the model. Any adjustment to this number could be compensated by small changes in synaptic weights, as demonstrated by our simulations.
   We have changed the text in the supplementary information section "Neuron model derivation" accordingly: "We used the following neuron parameters: $\tau_m = 1.5\,\text{ms}$. Furthermore, $\tau_{ref} = 9\,\text{ms}$, $U_{res} = -11\,\text{mV}$, and $U_{th} = 15\,\text{mV}$. As stated above, U_{res} and U_{th} are measured relative to the membrane resting potential of $\approx -65\,\text{mV}$. We chose the values of U_{th} and τ_{ref} so that the spike times of the integrate-and-fire model are as similar as possible to the spike times of a Hodgkin-Huxley simulation with the parameter values in Dayan &Abbott (2001)" (lines 924-929).

2. line 146, "inhomogeneous Poisson process" implies a time-dependent rate, so r_i (in the same line) should have a time-argument.

   We have made the suggested change, ensuring that r_i now includes a time argument to accurately reflect the time-dependent nature of the inhomogeneous Poisson process (line 957 and Equation (19)).

3. line 160: "angular speed neurons": Misleading term, as these neurons do not encode speed (—v—) but rather rectified velocities (max(0,v) and max(0,-v), respectively).

   We agree with your assessment that "velocity" is the more accurate term, as the neurons in question encode directional movement rather than just the magnitude. We have updated the manuscript to use "velocity neurons" instead of "speed neurons" to better reflect this distinction."

4. line 333: The model performance was calculated based on the true and estimated heading directions after some fixed time period. However, a small mismatch at the end of the experiment does not imply a small mismatch throughout the entire time course – the system might have drifted by 360°. I don't think that this happens (often), but one nevertheless has to check the possibility of such an error.

   While we agree that such drift is conceivable, we have not encountered this issue in our simulations. The supplementary Figure S1 shows the

development of the error over time. As this figure demonstrates, the agent maintains a stable heading estimate over a longer period, and any potential 360° drift would be apparent within the intervals examined.

5. line 351ff – agent simulation: I miss the characterization of the path to be followed (straight or stochastic changes of velocity or direction, etc).

   In the agent simulation, the goal is to travel in a specific direction rather than adhering to a predefined path. We do not control velocity or introduce stochastic changes of direction during the simulation, but we perturb the agent by translational and rotational noise. The agent's ability to align with the target direction is evaluated by comparing its heading at the final point in time of the simulation to the ideal heading direction that matches the goal direction. As stated in the manuscript: "The ability of the agent to maintain a steady travel direction was quantified by Equation (9), with ϕ and ϕ' as the actual and the ideal heading direction (matching the goal direction) of the agent at the final point in time of the behavioural simulation" (lines 434-436). This approach provides a measure of the agent's accuracy at the temporal endpoint but does not track its alignment throughout the entire simulation period.

6. line 496: I don't understand the statement that a "weight noise up to 5 % only marginally increased the number of larger errors in the heading estimate" – for a 5 % noise level, the heading distribution is essentially flat (see Fig 5C)!

   We have revised the statement to clarify: "Membrane noise up to 1 mV only marginally increased the number of larger errors in the heading estimate (cf. Fig 5B). In contrast, even small amounts of weight noise have a detrimental effect on integration performance (Fig 5C)" (lines 555-552). This distinction emphasizes the different impacts of membrane and weight noise on our model's performance.

7. line 531: Figure S5A-B has a poor color code and one cannot really see what the authors want to show. In addition, I could not find information on the rather large temporal discretization (5 s).

   We recorded the angular deviations every 5 s along the generated agent trajectories. We have added a statement to this effect in the figure caption for supplementary Figure S1 (previously labeled S5): "Mean-squared deviation between the agent's heading estimate and the ground truth heading direction, averaged over 1000 trials lasting 20 s each, recorded every 5 s. Panels A and B demonstrate the ability of the agent to maintain straight-line orientation under conditions with different probabilities of being translated (A) or rotated (B) by wind. Solid lines show the mean angular deviation, dashed lines are mean ± one standard deviation. For details, see text."
   We have further updated Figure S1 (previously titled S5) by using out-

lined curves instead of filled areas to improve the discernibility of results across different probabilities of rotation and translation.

8. line 532: the statement "without (A) and with (B) wind-induced perturbations" does not fit with what is shown in the figure.

   We have adjusted the text in the "Accuracy and robustness of heading and angular velocity integration" subsection of the "Results" section accordingly: "Supplementary Figure S1A and B demonstrate the ability of the agent to maintain straight-line orientation under conditions with different probabilities of being translated (A) or rotated (B) by wind" (lines 597-600).

9. line 532/533: The statement "These results demonstrate the simulated agent's capability to carry out goal-directed locomotion in dynamic environments..." seems to need an explanation as the angular mismatch is typically at least 10° (7C, 7D).

   We have adjusted the text in the "Accuracy and robustness of heading and angular velocity integration" subsection of the "Results" section accordingly to better reflect the results, as follows: "Together, these results demonstrate the simulated agent's capability to carry out goal-directed locomotion in the presence of environmental perturbations. Notably, rotational perturbations can improve straight-line navigation: the bimodal error distribution in Figure 7 becomes more unimodal with increasing rotational perturbations. This is likely due to the larger error signals that are available under rotationally perturbed conditions compared to either unperturbed conditions or translation-only perturbations." (lines 605-610).

10. line 534ff (Caption of Fig 7): Why is the distribution of angular deviations bimodal?

    The bimodal distribution of angular deviations observed in Fig. 7C-D reflects that the system frequently produces deviations from the goal direction to the left or right but seldom remains perfectly aligned (at 0° deviation).
    We have updated the text in the "Accuracy and robustness of heading and angular velocity integration" subsection of the "Results" section to clarify this interpretation: "The distribution of angular deviations is bimodal, indicating that the system frequently experiences minor deviations to the left and right from the goal direction but reaches exact zero deviation less often. Supplementary Figures S1A and B demonstrate the ability of the agent to maintain straight-line orientation under conditions with different probabilities of being translated (A) or rotated (B) by wind. These panels demonstrate that left and right errors occur equally often and that overall error decreases over time, suggesting that these deviations tend to cancel each other out if they don't systematically accumulate in one direction. Importantly, these minor deviations are part of the system's adaptation

to dynamic conditions and do not significantly affect the agent's overall ability to maintain goal-directed locomotion." (lines 594-605).

11. line 534ff (Caption of Fig 7): How many time steps do 12.5 s correspond to?

    An agent time step is 0.1 s long, so 12.5 s corresponds to 125 agent steps. The circuit is integrated in 1 ms increments.

    To improve clarity, we have added this information at three places in the manuscript:

    (a) In the caption of Figure 7: "In all simulations, an agent time step is 0.1 s long, and the heading circuit is integrated in 0.001 s increments".
    (b) In the "Free parameters and their optimisation" section of the "Results" section: "We used 4 ms integration time steps for the optimisation of free parameters and 1 ms for all simulations reported here" (lines 317-318).
    (c) In the "Testing of the heading circuit in an agent simulation" subsection of the "Results" section: "(an agent time step is 0.1 s long)" (line 441).

12. line 687: "Panels A and B demonstrate the impact of the probability of a wind gust..." Shouldn't it read "Panels A and B demonstrate the impact of a wind gust with a certain the probability ..."? (a wind gust might have an impact but not its probability)

    You are right, the updated sentence in the caption for Supplementary Figure S1 now reads: "Panels A and B demonstrate the ability of the agent to maintain straight-line orientation under conditions with different probabilities of being translated (A) or rotated (B) by wind".

13. line 715, Equation (15): Shouldn't the "log" be a "ln"?

    Yes, we have corrected Equation (15) accordingly.

14. lines 717 and 721: $\mathrm{V_{reset}}$ should be $\mathrm{U_{res}}$

    We have corrected the notation to use $\mathrm{U_{res}}$ instead of $\mathrm{V_{reset}}$ (lines 920 & 925).

15. line 719/720: "deviations whose average effect can be well described by a logistic sigmoid" a reference for this statement would be appreciated.

    We have updated the supporting information subsection "Neuron model derivation" (lines 934-937 and Supplementary Figure S2) to argue for a logistic sigmoid approximation to the rate function:

    "However, membrane potential fluctuations will lead to deviations whose average effect can be well described by a logistic sigmoid:

$$r(U_\infty) \approx \frac{A}{1 + \exp(-B \cdot (U_\infty - C))} \quad (1)$$

We use the following neuron parameters: $\tau_m = 1.5\,\text{ms}$ which can be derived from the resting state conductance of a Hodgkin-Huxley model with parameters given in [Dayan & Abbott, 2001]. Furthermore, $\tau_{ref} = 9\,\text{ms}$, $U_{res} = -11\,\text{mV}$, and $U_{th} = 15\,\text{mV}$. As stated above, U_{res} and U_{th} are measured relative to the membrane resting potential of $\approx -65\,\text{mV}$. We chose the values of U_{th} and τ_{ref} so that the spike times of the integrate-and-fire model are as similar as possible to the spike times of a Hodgkin-Huxley simulation with the parameter values in [Dayan & Abbott, 2001]. For random fluctuations with a standard deviation of 8 mV, which has been observed in locust neurons (see e.g. Moreaux et al. (2007), Fig. 2), the best fitting sigmoid parameters are:

$$A = 99.6\,\text{Hz},\ B = 0.19\,\text{mV}^{-1}\ C = 17.8\,\text{mV}. \tag{2}$$

These values can be reproduced by running the script `invertpy/brain/centralcomplex/cl1acl2/fit_-rate_function.py` in the repository." This script will also output supplementary Figure S2, which shows the logistic sigmoid approximation to the average rate function with 8 mV membrane noise.

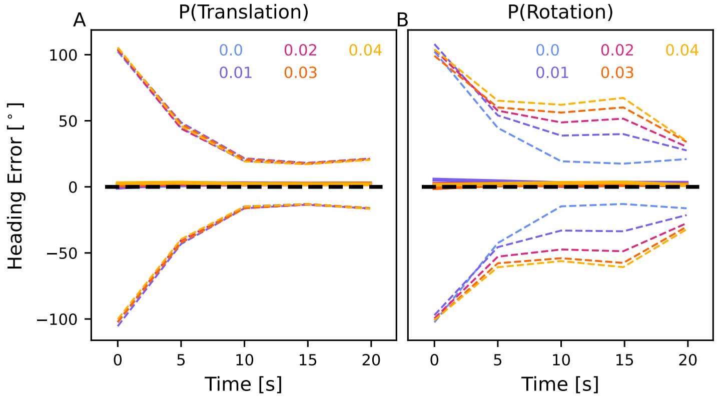

Sigmoid rate function approximation. Green dotted line: rate function of the ideal integrate-and-fire neuron without noise. Blue dashed line: rate function with $\sigma = 8\,\text{mV}$ membrane noise, averaged across 1000 simulations. Solid orange line: best fit obtained with a logistic sigmoid. For details, see text.

16. line 721/722: The authors list parameter values "which are typical values for cortical neurons" and cite the textbook by Dayan and Abbott (2001) to support this statement. I could not find these parameter values in Dayan and Abbott (2001).

    We have adjusted the "Neuron model" subsection of the "Model and methods" section to clarify: "While $\tau_m = 1.5\,\text{ms}$ can be derived from the resting state conductance of a Hodgkin-Huxley model with the parameter values given Dayan & Abbott 2001,the membrane time constants used in the

fruit fly CX model of Turner-Evans et al. (2017) are larger by an order of magnitude to capture observed delays between E-PG- and P-EN-neuron activity in walking flies (Turner-Evans et al, 2017)" (lines 148-152). For the other parameters, please refer to the replies above, which include relevant simulations.

17. line 723: I do not understand the argument leading to the assumption that thermally induced voltage fluctuations are around 8 mV. Given the authors' own argument, they should rather be around 25 mV (which clearly shows that the authors' logic does not hold). In this context, reference 84 appears to be of little help as it deals with an in- vivo preparation where synaptic inputs might be the main cause of the observed voltage fluctuations. Finally, theoretical work by Steinmetz et al. (J Comp Neurosci 2000) shows that thermal fluctuations are typically well below 2 mV.

    Thank you for pointing out this discrepancy. Thermally induced fluctuations are in the order of 25 mV per singly charged particle, and can be expected to be smaller on average. We would argue that the source of the fluctuations – thermal or pre-synaptic – is not very relevant for our argument.
    We have adjusted the text in the supporting material section 'Neuron model derivation' accordingly: "For random fluctuations with a standard deviation of 8 mV, which has been observed in locust neurons (see e.g. Moreaux, 2007, Fig. 2), the best fitting sigmoid parameters are..." (lines 929-933). However, even if the fluctuations are as little as 2 mV, a logistic sigmoid with suitably chosen parameters is still a good fit for the average rate function. We invite you to run the script `invertpy/brain/centralcomplex/cl1acl2/fit_rate_function.py` in the repository to reproduce this figure:

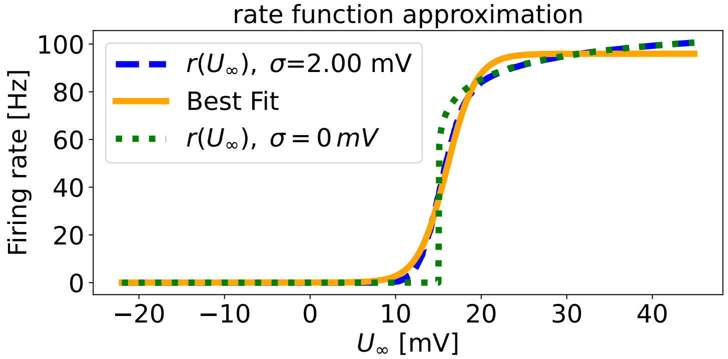

18. line 727: Average rate functions might have a sigmoid shape. This does, however, not provide any justification for the specific parameter values.

    To address your concern about the specific parameter values, we invite you to review the fit results produced by the script `invertpy/brain/centralcomplex/cl1acl2.fit_rate_function.py`. This script performs the fitting of a logistic sigmoid to

the average noisy rate function of an integrate-and-fire neuron across various noise levels and computes the sigmoid values used in the manuscript. It will output the following figure along with the used parameter values:

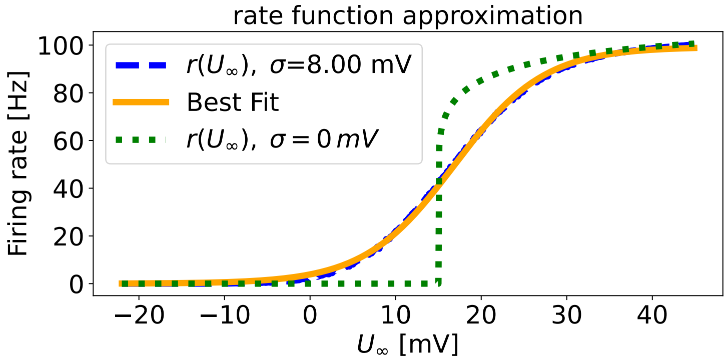

As shown in the image, the parameter values yield a very good fit with the data. We have also added this figure and its description to the supporting information subsection "Neuron model derivation" as Supplementary Figure S2. Additionally, the simulations referenced in our previous replies regarding the relationship between the membrane time constant and refractory period demonstrate that the precise sigmoid parameter values are not crucial for our results. We find that good angular velocity integration is achievable with a range of sigmoid parameters.

19. line 754ff: I may miss a crucial point, but I simply do not understand how a synapse with fixed reversal potential can be both, excitatory and inhibitory.

    In our model, we used a fixed reversal potential representing the typical potential for cation channels, and whether a synapse is excitatory or inhibitory is determined by sign of the synaptic conductance.
    We have added an explanation to the "Neuron model" subsection of the "Model and methods" section: "In our model, the sign of the synaptic weight determines whether a synapse is excitatory or inhibitory (positive or negative synaptic weight, respectively)" (lines 171-173).
    Further, we have adjusted the "Synapse model" subsection of the "Neuron model" section of the Supplementary Information: "Since we did not know a priori whether a synapse would be inhibitory or excitatory, we fixed the synaptic reversal potential of each synapse i at $E_{s,i} = 65\,\mathrm{mV}$. This is approximately the average reversal potential for cations (Na^+, K^+) relative to the membrane resting potential (Kandel, 2000). We allowed for both positive (excitatory) and negative (inhibitory) synaptic weights $\tilde{g}_{s,i}$ and $\tilde{g}_{mod,i,j}$" (lines 998-1004).

20. In addition, I would like to see a specific reference for the statement that 65 mV "is approximately the reversal potential for cations relative to the resting potential.

We have added a reference to the "Neuron model" section of the supplementary information: "...we fixed the synaptic reversal potential of each synapse i at $E_{s,i} = 65\,\text{mV}$. This is approximately the average reversal potential for cations (Na^+, K^+) relative to the membrane resting potential (Kandel et al., 2000)" (lines 999-1001).

21. Finally, Equation (22) does not seem to be compatible with Equation (13).

    Equation (22) is Equation (13) with $\tilde{g}_{tot,i}$ (as defined in Equation (20)) substituted for $\tilde{g}_{s,i}$. To make this clearer, we now introduce Equation (22) via "By substituting $\tilde{g}_{tot,i}$ for $\tilde{g}_{s,i}$, the steady-state potential (Equation (13)) becomes: [...]" (line 1003-1004).

22. line 764ff: "... P-EN activity precedes P-EN activity in the EB." There is something wrong or missing.

    The sentence has been corrected to reflect the accurate sequence of activity: "P-EN activity in the EB leads E-PG activity" (lines 1009-1010).

23. line 781: "... transmission delay between E-PG and P-EN ..." Shouldn't it be the other way round?

    It should indeed be the other way round. We have now corrected this in the manuscript accordingly: "Let the unknown transmission delay between P-EN and E-PG be Δt" (lines 1027-1028).

24. line 785: one "P-EN" should be an "E-PG".

    We have corrected the sentence to read: "In the first experiment, GCaMP6f is expressed in P-EN-neurons, and jRGECO1a is expressed in E-PG-neurons" (lines 1031-1033).

25. line 792: Why does a long transmission delay justify "the use of a steady-state neuron model"? Typically, delay-differential equations with large negative feedback show oscillations.

    We assume that the membrane has a shorter time constant than synapses, which basically acts as a low-pass filter on the pre-synaptic input. We now elaborate in the supplementary information section "Neuron model derivation": "Precise values for τ_s in the locust are currently unknown, but drawing on our estimates from available fruit fly data, we argue for moderately slow synapses (see section 'Functional transmission delays between CL1a- and CL2-neuron populations'). We chose $\tau_s = 20\,\text{ms}$. This choice provides a balance between two important factors. First, it helps smooth out noise in angular velocity integration by acting as a low-pass filter. Second, it allows the compass representation to respond effectively to angular velocity inputs, within the range we have modeled: $[-150\,°/\text{s}, 150\,°/\text{s}]$." (lines 948-954).
    We have not observed oscillatory behaviour in our solutions. This may be due to the weights that arise after the optimization, which promotes

non-oscillatory solutions, as stated in the "Free parameters and their optimisation" subsection of the "Model and methods" section: "All synaptic weights were optimised so that the network reproduced activity targets encoding the true heading during or after an angular velocity integration time interval of 200 ms" (lines 310-312).

26. Figure 7A: Why does the agent not improve its heading direction with increasing time? Shouldn't it be able to compare its heading direction with the goal direction? And why do the added perturbations in 7B improve the situation?

    We have added the following explanation to the "Accuracy and robustness of heading and angular velocity integration" subsection of the "Results" section: "In Figure 7A, the agent's heading did not improve after an initial approximate alignment with the goal direction. This may be due to a residual heading error that is too small to be corrected. In contrast, Figure 7B shows how the introduction of wind perturbations provides more pronounced feedback that helps the agent adjust its heading direction more effectively towards the goal" (lines 583-588).

2 Review B

The manuscript by Pabst et al. puts forward a computational model for angular velocity integration in the central complex of the locust. The proposed model is noteworthy in that it differs significantly from previous models grounded in experimental data from Drosophila. Whereas experiments in flies indicate that each side of the protocerebral bridge represents the full 360° of azimuthal space, the proposed model assumes that each side of the bridge represents only 180o based on intracellular recordings performed in the locust. In my view, the present study is of value because the hypothesis that the protocerebral bridge of the locust only represents 360° is intriguing—computational studies that investigate the plausibility of this hypothesis (or lack thereof) could provide concrete predictions for experimentally testing this model or prompt a re-evaluation of the data that originally inspired it. Overall, I found that the manuscript would greatly benefit from improvements in clarity, in particular with regards to providing the reader with an intuition for how the model works. I also think that the biological plausibility of the proposed model, as well as experiments that could test it, should be discussed more.

2.1 Major points

1. It is not clear how the dynamics in CL1a/CL2 connectivity actually cause the bump of activity in the bridge to be updated during turns. In the Drosophila heading system, the imbalance in activity between left and right PEN neurons and the anatomical shift from the bridge to the ellipsoid body are two key features for updating the position of the EPG

bump of activity. From my understanding, neither of the features play a role in updating the position of the EPG/CL1a bump of activity in the proposed model. Instead, the authors posit the existence of some neuron that modifies the synaptic weights of the CL1a and CL2 neurons via some neuromodulator depending on the insect's angular velocity. Presumably differences in the activity of CL2 neurons somehow cause the CL1a bump to move, but how exactly was not clear from Figure 4 (I did not find the force- directed graphs useful). The authors should explain this more clearly. Perhaps a cartoon highlighting the key connections that are important for updating the CL1a bump position during turns and how they are dynamical modified would be helpful.

We have updated the "A proposed circuit for heading and angular velocity integration in the desert locust CX" subsection of the "Results" section to provide a more intuitive explanation of the circuit properties allowing the circuit to update the position of the compass bump: "The core mechanism driving the shift of the activity bump during turns relies on neuromodulation of synapses between neurons arborizing in neighboring columns of the PB, as opposed to the feed-forward mechanisms found in the *Drosophila* heading system. The weight modulations during clockwise and counterclockwise turns are complementary — negative modulatory weights in Figure 4E are positive in Figure 4F and vice versa. These modulations, which are offset from the diagonals, induce asymmetries in the effective, modulated weight matrices (see Figures 4B and C). This directional bias allows excitation to propagate toward neighboring columns in one direction versus the other, shifting the activity bump in tune with the animal's turn direction" (lines 472-487).
We have further updated the "Model constraints and properties" subsection of the "Discussion" section to provide a more intuitive understanding of the dynamics underlying the bump shift, and to compare our model with the established fruit fly heading circuit model: "The fruit fly model by (Turner-Evans et al., 2017) posits asymmetric feed-forward angular velocity inputs to the two halves of the PB that additively modulate firing rates in the heading circuit. Instead of relying on such feed-forward inputs, our model features neuromodulatory angular velocity inputs that effectively phase-shift the heading signal through multiplicative modulation of firing rates (Zhang, 1996), cf. Equation (10). While our model focuses on synaptic-level neuromodulation, other mechanisms could also achieve multiplicative effects. For instance, multiplication can occur through exponentiation by active membrane conductances of summed logarithmic inputs, nonlinear dendritic interactions, or network dynamics (Issa & Zhang 2012). An anatomical offset between the projections of E-PG- and P-EN-neurons in the PB and EB of *Drosophila* has been proposed to facilitate activity bump shifts across these regions in both theoretical and computational models (Green et al., 2017; Turner-Evans et al., 2017). A similar offset is conserved across species (Sayre et al. 2021) and is mirrored in

our circuit's connectivity (see Figure 2 for the CL1a and CL2 projection schemes and their derived connectivity). However, because our model employs single-compartment neurons, we do not explicitly separate neuron endings in different neuropils of the CX. Exploring this anatomical distinction and its effects on compass bump dynamics will be a key focus for future modeling efforts." (lines 649-666).
We have also updated Figure 4 G and H to highlight the effect of angular velocity inputs on a subset of the synaptic connections in the circuit, instead of showing the circuit in its entirety during zero angular velocity.

2. On line 584 the authors highlight that it is unclear whether a 360° model of the protocerebral bridge is compatible with recurrent inputs from TB1/2 neurons (Delta7 neurons in flies). This is a very good point. The fact that individual TB1/2 neurons have outputs in the bridge every 8 glomeruli would seem to imply that the preferred heading angles should be similar, or the same, every 8 glomeruli, which is inconsistent with the 360° model of the bridge. I think this should be addressed in the present paper by incorporating these connections in the proposed model.

   We agree that further exploring these connections could provide valuable insights. However, the primary focus of this manuscript is to explore and validate the core principles of heading direction representation, angular velocity integration and steering in the current model framework.
   We optimized the CL1a → TB1 connectivity in our model to provide the steering model with a single-bump heading representation. Adding recurrent TB1 → Cl1a connections and re-running this optimization would set the synaptic weights of these recurrent connections close to zero, because they are not needed for the steering objective. In addition, while TB1/2 neurons do project outputs every 8 glomeruli, it is important to consider that these neurons may not supply inputs to all neural populations in the PB equally. Specifically, the population we are modeling might not receive inputs from TB1/2 neurons at each of these output sites. This suggests a more selective connectivity pattern, where certain populations contribute to a 360° heading representation while others serve distinct functional roles.
   Without further empirical data, it is difficult to ascertain the exact contribution of TB1/2 inputs to the population encoding heading direction across the full width of the PB. We acknowledge that integrating these recurrent connections into the model would provide a more complete picture of the overall circuitry and are keen to explore this avenue in future work. This will allow us to investigate how TB1/2 inputs may refine or modulate heading direction representations and whether they impose constraints on the 360° model.

3. The authors should propose experiments that could be performed by future studies to rigorously test their model. For example, does their model make any predictions about the functional properties of CL2 and CL1a

neurons that are not expected based on the Drosophila heading system?

We have added a subsection "Predictions and open questions for future research" to the "Discussion" section: "In the previous discussion, we proposed several avenues for future experimental investigations aimed at rigorously testing our model of the locust heading circuit. Below, we summarize the key predictions and open questions derived from our model and its implications:

1. **Investigating model connectivity via electron microscopy:** We anticipate that detailed electron microscopy studies of CL1a- and CL2-neurons as well as tangential neurons will uncover additional synaptic connections within the heading circuit of the desert locust, refining the connectivity assumptions underlying our model.
2. **Assessing temporal dynamics alignment:** Future experiments, e.g. simultaneous pre- and postsynapic intracellular recordings, should determine whether the temporal dynamics of neurotransmitter release and modulation align with our model's behaviour.
3. **Examining the role of tangential neurons in angular velocity integration:** We expect physiological studies to show that TB-, TL-, or TNL-neurons respond to rotational cues, comparable to GLNO-neurons in the fruit fly (Hulse et al., 2023). Further functional studies of the modulatory neurotransmitters detected in these tangential neurons (von Hadeln et al., 2020) could validate their roles in our model of the locust heading circuit.
4. **Role of TB-neurons:** We predict that including TB1- and TB2-neurons in future models will reveal significant contributions to heading representation, enhancing our understanding of their potential role as intermediaries in the circuit.
5. **Implementing spiking neuron models:** We anticipate that spiking neuron models will replicate the principles of heading and angular velocity integration implemented in our model. This would confirm our model's applicability to biological systems and underscore neuromodulation as a potential mechanism for introducing asymmetries into heading circuits (Zhang, 1996).
6. **Testing lead-lag relationships between CL1a and CL2 activity bumps:** Future circuit models employing multi-compartmental neurons should demonstrate reversed lead-lag relationships in the activity of CL1a- and CL2-neurons between the PB and CBL, mirroring findings from E-PG- and P-EN-neurons in the fruit fly (Turner-Evans et al., 2017).
7. **Determining synaptic delays:** We expect that experiments on reaction times to shifting visual target stimuli in locusts will yield upper bounds for our assumptions about synaptic delays. Two-photon

calcium imaging studies of CL1a- and CL2-neurons in locust, as performed with E-PG- and P-EN-neurons in fruit flies (Turner-Evans et al., 2017), would be crucial for exploring species-specific differences in integration and firing dynamics of the involved neurons.

8. **Investigating low-pass filtering dynamics:** We predict that simultaneous neuro-stimulation and multicellular recordings at different positions in the PB will indicate that the CL2-neurons' activity is a low-pass filtered version of the CL1a-neurons' activity, when the latter are stimulated. Furthermore, it will be interesting to see if the time constants of this low-pass filter are comparable to *Drosophila*'s

9. **Assessing multimodal cue integration:** Simulating cue integration in both naturalistic and laboratory conditions should reveal how well the model performs under conflicting cues, allowing comparisons with studies of other insect species (Shaverdian et al., 2022; Mitchell et al. 2023).

10. **Incorporating sky compass cues:** We expect that integrating sky compass cues will enhance the accuracy of the model's heading estimate and its predictive capacity for natural heading updates. This would close the loop between environment, body, and brain (Chiel & Beer, 2009), allowing for simulations of experiments from both field and laboratory settings.

11. **Exploring integration across temporal and spatial scales:** Future research should investigate how different phases of navigation tasks are integrated, particularly focusing on which cues from a multimodal set are most relevant for orientation in each context (Mouritsen, 2001; Fiehler & Karimpur 2023).

" (lines 821-882).

2.2 Minor points

1. It would be helpful if Figure 1 also included the expected activity pattern in the CBL/EB since this a significant difference between the fly heading system and the proposed model. It seems likely that the absence of a coherent bump of activity in the CBL (i.e., the presence of two intercalated 180° representations) might pose additional challenges to the system.

   We have updated Figure 1 to include the distribution of preferred heading directions in the EB and CBL.
   Regarding the potential challenges posed by the two intercalated 180° representations in the CBL, our model and results suggest that this configuration does not introduce significant problems for the system's function. However, we acknowledge that this might differ in parts of the central complex not included in our current model. We appreciate your point and will consider this aspect in future iterations of our work.

2. Is it necessary for both the CL1a and CL2 synapses to be modulated by angular velocity for the model to work? Why did the authors choose to do this?

   While it is not strictly necessary to modulate both the CL1a and CL2 synapses for the model to function, doing so enhances integration accuracy slightly. We report in the manuscript: "To determine how much neuromodulation in the PB, CBL and/or NOL contribute to the accuracy of angular velocity integration, we restricted modulatory inputs to either the CL1a-neurons or the CL2-neurons and re-optimized the network. We evaluated the average absolute integration error and its standard error at the simulation endpoint after 2000 trials of 4 s duration (...) with a slightly higher accuracy for the CL1a+Cl2 modulated network" (lines 538-547).

3. On line 367 it says that in the model by Stone et al. 2017 "the current heading direction is represented by two compass bumps across 16 CL1a-neurons. This is transformed into a single bump across 8 TB1-neurons." This is confusing because the spatial pattern activity of TB1 neurons in the bridge consists of two bumps of activity in Stone et al. 2017 (see Figure 5h).

   Thank you for your observation. We believe Figure 5h from the paper by Stone et al. (2017) depicts each TB1-neuron twice to better illustrate the circuit connectivity. Notably, the TB1-neurons on the left and right share the same activity and labels, indicating they represent the same neurons. The text mentions only eight neurons ("Our model assumes that each of the eight multiglomerular TB1 neurons in the PB [...]"), which is also in line with the model code provided by the authors.

4. On line 455 the authors state that "the synaptic strengths are up- and down-regulated during turns, but the polarity of synapses (excitatory or inhibitory) is not altered by neuromodulation". However, the sign of the weights of several neurons in Figure 4B change in Figure 4C. Are these not synaptic weights? Please clarify.

   Thank you for catching this mistake and bringing it to our attention. You are correct that the synaptic weights in Figures 4B and 4C do change sign in certain cases. We have removed the statement regarding the polarity of synapses, as it no longer reflects the model in its current form.

   Additionally, we now propose potential biological mechanism to explain these functional changes in the discussion section "Neurobiological Plausibility": "These modulations may require more complex biological mechanisms than our model directly represents. For instance, it is plausible that different CL2-to-CL1a connections from the same presynaptic neuron could be modulated differently during the same turn, a process that might involve distinct neuromodulators or receptor-specific effects. This complexity could be achieved through spatially segregated synapses with different receptor expressions, or through co-transmission of neuromodulators with opposing actions, depending on the context and intracellular sig-

naling cascades. Such effects could biologically be implemented in tangential neurons innervating the PB (TB3-TB8-neurons), CBL (TL-neurons), and the lower unit of the NO (NOL, TNL-neurons), most likely through processes more complex than those described in our model. For example, additional interneurons could effectively turn up- into down-regulation" (lines 722-732).

5. In the proposed model, not only do the connections between a CL2 neuron and a CL1a neuron get up or down-regulated depending on whether the insect is turning clockwise or counter-clockwise, but also different CL2-to-CL1a connections (from the same pre- synaptic CL2 neuron) are modulated differently during a same turn. I believe these two features of the model requires two types of neuromodulators and differences in how these two neuromodulators are received. This was not clear in the Neurobiological plausibility section (which made it seem like only one of these features would be necessary).

   We have revised the relevant section in the discussion to clarify the potential mechanisms involved: "These modulations may require more complex biological mechanisms than our model directly represents. For instance, it is plausible that different CL2-to-CL1a connections from the same presynaptic neuron could be modulated differently during the same turn, a process that might involve distinct neuromodulators or receptor-specific effects. This complexity could be achieved through spatially segregated synapses with different receptor expressions, or through co-transmission of neuromodulators with opposing actions, depending on the context and intracellular signaling cascades. Such effects could biologically be implemented in tangential neurons innervating the PB (TB3-TB8-neurons), CBL (TL-neurons), and the lower unit of the NO (NOL, TNL-neurons), most likely through processes more complex than those described in our model. For example, additional interneurons could effectively turn up- into down-regulation. Another explanation could be that the up- or down-regulating effect of the same modulators depends on postsynaptic receptors or signal cascades. While the presence of neuromodulatory transmitters suggests potential for modulation, their effectiveness in our model relies on more than just their presence. Specifically, for the model to work as intended, these transmitters must be able to both up- and down-regulate synaptic strengths with appropriate timing. Long-lasting neuropeptides, for instance, may not be able to provide the rapid and reversible modulation required. Future experiments should focus on determining whether the temporal dynamics of neurotransmitter release and modulation align with the requirements of our model" (lines 722-742).

6. In Drosophila, the GLNO neurons provide rotational velocity inputs to PEN neurons (Hulse et al. 2023 `https://doi.org/10.1101/2023.09.25.559373`). Are the corresponding neurons in locusts (the TNL neurons) also tuned to optic flow? If so, would these inputs be compatible with the

proposed 360° model of the bridge?

We have made an update to the Introduction: "Recent studies have identified tangential GLNO-neurons as a source of rotational velocity information to P-EN-neurons in the fly. They rely primarily on motor signals but can alternatively use visual information (Hulse et al., 2023)", and "Tangential neurons homologous to GLNO-neurons have been termed TNL-neurons (Homberg et al., 2022), but data revealing their responsiveness to angular velocity are lacking" (lines 105-108 and 114-115).
We have further made updates to the Discussion, section "Neurobiological plausibility": "In fruit flies, GLNO-neurons have been identified as providers of rotational velocity information to P-EN-neurons, prioritizing motor signals while using visual information only under specific conditions (Hulse et al. 2023). Investigating whether analogous TNL-neurons in locusts (Homberg et al. 2013, von Hadeln et al. 2020, Homberg et al. 2022) perform similar functions could enhance our understanding of the evolutionary conservation of these circuits across insect species" (lines 751-756).

7. In the proposed model, does the bump of activity drift when the angular velocity is zero? This is somethings that was investigated by Noorman et al. 2022 (`https://doi.org/10.1101/2022.05.23.493052`).

   We have extended the "Free Parameters and Their Optimization" section of the "Model and Methods": "All synaptic weights were optimised so that the network reproduced activity targets encoding the true heading during or after an integration time interval of 200 ms angular velocity integration interval. In line with observed behaviour in the fruit fly head direction circuit reported by Noorman et al. (2022), these activity targets were defined to promote a stable heading representation with no drift when the angular velocity is zero" (lines 310-315).
