# Supplementary material for: A computational model for angular velocity integration in a locust heading circuit
Source: PLoS Comput Biol. 2024 Dec 20;20(12):e1012155. doi: 10.1371/journal.pcbi.1012155 (PMC11703117; doi:10.1371/journal.pcbi.1012155)
Supplement: Attachment — Submitted filename: Pabst_2024_review_round_2.pdf [file pcbi.1012155.s010.pdf]

I thank the authors for responding to each of the issues that I raised, as well as making changes to their manuscript to address them. The revised manuscript is noticeably clearer with regards to how the proposed model works. With the benefit of this added clarity, after my second reading of the manuscript I was left convinced that the biological plausibility of the proposed model is rather low. I was also disappointed by the authors attempt to propose experiments that would rigorously test their model. That said, because the model developed in this paper is intended as a proof of concept, I believe that neither of these issues should prevent the publication of this manuscript.

The proposed model stems from the assumption that the locust protocerebral bridge represents 360º of azimuthal space. This constraint, combined with the known anatomy of the central complex, poses several challenges to a circuit that integrates angular velocity to compute heading. In the proposed model, these problems are overcome using "dynamic synapses". The reason the proposed model seems biologically unlikely is therefore twofold. First, anatomical features that are present in the central complex of both locusts and flies, and have a clear functional purpose in *Drosophila*, are effectively erased by neuromodulation, as they only hinder the model, begging the question of why these anatomical features are present in locusts in the first place. Second, every anatomical feature that poses a problem to the model requires invoking additional neural mechanisms, for which there is no obvious precedent in the literature. Below, I have highlighted two of these anatomical features, but I expect that incorporating additional anatomical and physiological detail to the model will pose additional challenges:

1. Each TB neuron is anatomically poised to receive information from CL1a neurons that are spaced 8 glomeruli apart in the bridge. The Delta7 and EPG neurons of flies share a similar anatomy. In *Drosophila*, this anatomical feature has a clear functional purpose: the connectivity between EPG and Delta7 neurons is appropriate for transforming two bumps of activity in the bridge into a sinusoidal pattern of activity. In contrast, in the proposed model, this anatomical feature appears to be just a nuisance: CL1a-to-TB connections are modulated such that each TB neuron only receives information from a single glomerulus (Fig. 4D). How this precise pattern of modulation across the bridge would be achieved is not explained. If TB neurons were to instead receive inputs from all possible CL1a synapses (as in Fig. 3E), this connectivity would interfere with the presumed single bump of activity in the bridge, presumably causing the model to no longer work.
2. CL2 neurons have an anatomical shift in their projection pattern relative to CL1a neurons. This anatomical shift is also shared by the PEN neurons of *Drosophila* and serves a critical role for updating the EPG bump of activity. In the proposed model, this feature of the anatomy serves no apparent purpose. In fact, the authors note that updating the compass bump position can be accomplished without modulating CL2 synapses without a significant detriment to the system's ability to integrate angular velocity (there is only a 2º difference in the angular accuracy when comparing a model that modulates CL2+CL1a synapses versus CL1a synapses only). It is unclear to me

whether CL2 neurons are necessary at all within the proposed dynamic synapse framework of the paper, or whether they could be completely omitted from the model.

None of these issues are inherently problematic. It is possible that the anatomical features shared by flies and locusts serve different functions in locusts (although the authors do not offer any clues as to what these might be). It is also possible that the dynamic synapses required by the model might be implemented via neuromodulator signaling mechanisms with levels of spatial-temporal resolution that have not yet been described. However, I believe that a more parsimonious explanation is that the underlying assumption and motivation for the proposed model—that each side of the locust bridge represents only 180º of heading—might be wrong. Indeed, the authors themselves highlight that it is unknown whether the physiological findings suggesting a single bump of activity in the bridge would hold in walking or flying locusts. It is worth noting that in dangling flies, under certain experimental conditions, the EPG bump of activity can respond unexpectedly to an open-loop visual stimulus (see example fly #2 in Figure 3b of Fisher et al. 2022, https://doi.org/10.1038/s41586-022-05485-4, where the EPG bump of activity appears to be restricted to one half of the EB in response to a rotating bar).

The "*Predictions and open questions for future research*" section did not adequately address major point #3. None of the eleven points made by the authors provides a concrete and testable prediction of their model. For a prediction to be a useful test of their model, it should differ from what is expected from the alternative model (i.e., the *Drosophila* model). For instance, point #6 suggests that future studies should examine lead-lag relationships between CL1a and CL2 activity bumps. However, the authors do not explain how this lead-lag relationship is expected to differ from what has been previously measured in flies. If their model predicts the same lead-lag relationship, then this prediction is not a useful one. Or, for example, point #1 suggests that *"detailed electron microscopy studies of CL1a- and CL2-neurons as well as tangential neurons will uncover additional synaptic connections within the heading circuit of the desert locust, refining the connectivity assumptions underlying our model"*. Again, the authors do not provide any concrete predictions. What connectivity patterns are expected to be discovered based on their model, but are not expected based on *Drosophila* connectomic studies? For example, I could imagine that, given that some CL1a to TB synapses are completely silent in their model, and appear to have no functional purpose, that the authors might expect electron microscopy studies to reveal that these synapses are actually absent in locusts. In other words, despite TB neurons having dense dendritic arbors every 8 glomeruli, they would only synapse with CL1a neurons in one section of the bridge (as in Fig. 4D). This prediction would be completely unexpected from previous models, and would differ completely from what is observed in *Drosophila*. But perhaps the authors do not share this expectation?

I recognize that coming up with testable predictions of a model, especially ones that are experimentally feasible to test, is a difficult task. There can be more than one way to implement a model, making it hard to provide predictions that are strictly necessary for a model to work. Still, I was left with the impression that the authors did not make a genuine effort to propose experiments that will test their model. I strongly encourage the authors to revise this section of

their manuscript to come up with at least one concrete and testable prediction, that is specific to their model.

Despite my above comments, I believe that the central claim of the paper, that the "*[…] study supports the possibility that similar computations for orientation may be implemented differently in the neural hardware of the fruit fly and the locust"* still holds; the proposed model is not biologically impossible, it just seems highly unlikely. I also realize that evaluating the biological plausibility of the model is somewhat a matter of opinion, and it should perhaps be left to the reader to make their own judgement. For these reasons, I nonetheless support publication of this manuscript.
